# Effects of sesame (*Sesamum indicum* L.) and bioactive compounds (sesamin and sesamolin) on inflammation and atherosclerosis: A review

**DOI:** 10.1002/fsn3.3407

**Published:** 2023-05-22

**Authors:** Elham Hadipour, Seyed Ahmad Emami, Niloufar Tayarani‐Najaran, Zahra Tayarani‐Najaran

**Affiliations:** ^1^ Department of Biology, Faculty of Science University of Guilan Rasht Iran; ^2^ Department of Traditional Pharmacy, School of Pharmacy Mashhad University of Medical Sciences Mashhad Iran; ^3^ Department of Dental Prosthesis, School of Dentistry Mashhad University of Medical Sciences Mashhad Iran; ^4^ Targeted Drug Delivery Research Center Pharmaceutical Technology Institute, Mashhad University of Medical Sciences Mashhad Iran

**Keywords:** atherosclerosis, inflammation, sesame, sesame Lignans

## Abstract

Inflammation, oxidative stress, obesity, infection, hyperlipidemia, hypertension, and diabetes are the main causes of atherosclerosis, which in the long term lead to hardening of the arteries. In the current study, we reviewed recent findings of the mechanism of sesame and its active compounds of sesamin and sesamolin regulates on atherosclerosis. Sesame can decrease the lipid peroxidation and affect the enzymes, which control the balance of oxidative status in the body. Besides modulating the inflammatory cytokines, sesame regulates the main mediators of the signaling pathways in the process of inflammation, such as prostaglandin E2 (PGE2), nuclear factor kappa light‐chain enhancer of activated B cells (NF‐kB) and peroxisome proliferator‐activated receptor gamma (PPAR‐γ). Sesame decreases the growth of different pathogens. It fights against obesity and helps to reduce weight, body mass index (BMI), waist circumference, and lipid count of serum and liver. In addition to lowering fasting blood sugar (FBS), it decreases the hemoglobin A1c (HbA1c) and glucose levels and improves insulin function. With high content of linoleic acid, α‐linolenic acid, and total polyunsaturated fatty acid (PUFA), sesame efficiently controls the blood plasma lipids and changes the lipid profile. In the case of hypertension, it maintains the health of endothelium through multiple mechanisms and conserves the response of the arteries to vasodilation. PUFA in sesame suppresses blood clotting and fibrinogen activity. All the mentioned properties combat atherosclerosis and hardening of blood vessels, which are detailed in the present review for sesame.

## Graphical abstract

Inflammation, oxidative stress, obesity, infection, hyperlipidemia, hypertension, and diabetes are the main causes of atherosclerosis. We reviewed recent findings of the mechanism of sesame and its active compounds sesamin and sesamolin on the arthrosclerosis.

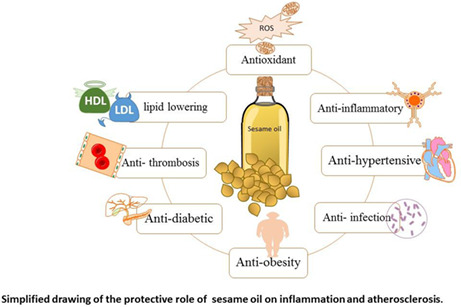

## 1 INTRODUCTION

The use of natural diet and herbs has become very popular in recent decades due to tendency for the consumption of toxin‐free food with minimal adverse effect (Kelly et al., 2005). Sesame seeds have the highest oil content among other seeds and are a common ingredient in various foods due to their unique flavor and aroma. Sesame has long been used as a popular edible grain in the food industry of Asian countries in various forms such as edible oil, cake batter, flour, and snacks with nuts (Fukuda et al., 1986a, 1986b; Gebremeskel et al., 2022; Hashempour‐Baltork et al., 2018; Namiki & Kobayashi, 1989; Shah, 2016). The genus *Sesamum* is one of the flowering plants and belongs to the *Pedaliaceae* family. This genus comprises about 20 species, most of which are indigenous to tropical Africa. Sesame was used in ancient Chinese civilization to obtain energy, calm the mind, and prevent aging (Weiss, 1983). Sesame helps to prevent a number of diseases such as hypertension, hypercholesterolemia, cancer, and aging (Kanu et al., 2010). It has also been shown to decrease the triglyceride (TG) and arachidonic acid (AA) levels, and also has antiinflammatory and estrogenic activities (Hemalatha, 2007; Shahidi et al., 1997). Countless phenolic compounds, including simple phenols and flavonols, are known to protect the heart. Sesame seeds, along with protein and lipid, contain lignans with phenolic compounds, such as sesamin and sesamolin, which are reported as valuable compounds for human health (Dalibalta et al., 2020; Fasuan et al., 2018; Jayaraj et al., 2020; Saleem et al., 2021).

Atherosclerosis is caused by thickening and hardening of the walls of the arteries and loss of their elasticity and the lesion called atheroma protrudes inside and leads to blockage of the vascular duct and the media below it. Atherosclerotic lesions begin with calcification of lipid‐filled foam cells, and then progress to lipid deposit, atheroma, fibroatromas, and eventually lead to complicated lesions. Important and effective factors in its progression to atheroma lesions are infection, obesity, hypertension, hyperlipidemia, diabetes mellitus, inflammatory process, and oxidative stress (Khan & Jackson, 2018; Rahman, 2001). The seeds and oil of sesame are resistant to oxidative conditions (Fukuda et al., 1986a, 1986b). Various studies have shown that sesame lowers oxidative stress, lipid peroxidation, blood pressure, blood lipids, and blood cholesterol, and also has antiproliferative activity (Ashakumary et al., 1999; Lee et al., 2005; Nakano et al., 2007; Suja et al., 2004; Visavadiya & Narasimhacharya, 2008; Yokota et al., 2007). The present review summarizes the effect of sesame and its famed lignans (sesamin and sesamolin) against inflammation and atherosclerosis. Related articles with keywords containing sesame or sesamin or sesamolin with atherosclerosis or inflammation in PubMed and Scopus have been reviewed up to September 2022.

## 2 PHYTOCHEMISTRY OF SESAME

Sesame is one of the most important healthy foods that has both nutritional and bioactive contents. Sesame contains a high amount of protein, fatty acids, vitamins, minerals, carbohydrates, phytosterols, and lignans (Esmaeilzadeh Kenari & Razavi, 2022; Prakash & Naik, 2014).

### 2.1 Nutritional profile of sesame

Sesame seeds are composed of 50%–52% of fatty acid glycerides (palmitic and stearic as saturated fat and oleic, linoleic, and linolenic as unsaturated), 17%–19% protein (arginine 140 mg, leucine 75 mg, methionine 36 mg, lysine 31 mg, and cysteine 25 mg), carbohydrates 16%–18% carbohydrates (3.2% glucose, 2.6% fructose, and 0.2% sucrose). Additionally, it contains about 10.8% fiber, minerals, vitamins, and phytosterols (Kheirati Rounizi et al., 2021; Prakash & Naik, 2014). Table 1 shows the nutritional profile of sesame (Table 1).

**TABLE 1:** Nutritional profile of sesame seed (per 100 g).

Nutritional profile of sesame	Amount
Protein	17 g
Carbohydrate	25.7 g
Fiber	14 g
Saturate fat	6.7 g
Monounsaturated fat	18.1 g
Polyunsaturated fat	21 g
Thiamin	0.8 mg
Riboflavin	0.3 mg
Niacin	4.6 mg
Vitamin B6	0.8 mg
Vitamin E	57.375 μg
Folate	98 mg
Carotene	60 μg
Calcium	989 mg
Iron	14.8 mg
Magnesium	356 mg
Phosphorus	638 mg
Potassium	475 mg
Zinc	7.2 mg
Copper	2.5 mg
Manganese	2.5 mg
Selenium	5.8 μg
Phytosterols	400–413 mg

### 2.2 Bioactive constituents of sesame

#### 2.2.1 Sesame lignans

Lignans are a group of low‐molecular weight polyphenols found in plants and are among the major compounds present in the phytoestrogens family. Lignans are relatively simple polyphenolic materials derived from phenylalanine and have oxidizing properties. Their main skeletons are 2, 3‐benzylbutane, and from the b–b junction, two structural units of C6C3 are formed. Oxidizing enzymes and dirigent protein control this conversion (Umezawa, 2003; Willför et al., 2006).

Sesame seeds contain both water‐soluble glucosides, lignans, and fat‐soluble lignans. Sesamin and sesamolin are fat‐soluble lignans and sesaminol triglucoside, sesaminol diglucoside, and sesaminol monoglucoside are soluble in water (Bedigian & Harlan, 1986; Budowski & Markley, 1951; Hemalatha, 2004). The structural difference between sesamin and sesamolin is due to the replacement of oxygen between the furofuran and piperonyl groups (Jeng & Hou, 2005). The amount of sesamin and sesamolin in sesame seeds is 200–500 mg/100 g and 200–300 mg/100 g, respectively (Kamal‐Eldin & Appelqvist, 1994). Also, the level of sesamin and sesamolin in sesame oils from roasted sesame seeds is 5–500 mg/kg and 5–500 mg/kg respectively (Liu et al., 2019). Lignans play an important role in protecting the sesame plant against pests in the form of powerful antioxidants and insecticides (Jeng & Hou, 2005). Sesamine and sesamolin have higher antioxidant effects due to the fact that they have four groups of OH compared with sesamol which has two groups of OH (Jeng & Hou, 2005). Sesamin is the most abundant lignan in roasted sesame seeds and sesame oils from roasted sesame seeds have the beneficial effects including antiinflammatory and antiallergic effects (Kamal‐Eldin & Appelqvist, 1994; Liu et al., 2019). It has also been shown to protect nerve cells from oxidative stress. In addition, it leads to the detoxification of chemicals and reduces the incidence of cancerous tumors caused by chemicals in the liver cell (Majdalawieh et al., 2021). It has also been shown that sesamin and sesamolin have antihypertensive effects (Nakano et al., 2007), increase the antioxidant activity of vitamin E in the lipid peroxidation system (Hemalatha & Rao, 2004), lower cholesterol (Visavadiya & Narasimhacharya, 2008), raise the oxidizing enzymes of fatty acids in the liver (Ashakumary et al., 1999), and protect neurons against hypoxia and brain damage (Cheng et al., 2006; Lee et al., 2005).

#### 2.2.2 Biosynthesis of lignans in sesame

In the biosynthesis pathway of sesamine, E‐Coniferyl alcohol is produced from the amino acid phenylalanine. It then leads to the production of pinoresinol in sesame seeds. Pinoresinol is converted to piperitol and sesamin in mature seeds by the CYP81Q1 gene. In younger seeds, pinoresinol is converted to sesamolin (Jiao et al., 1998; Ono et al., 2006).

## 3 PHARMACOKINETICS OF SESAME

Lot of work has been done to clarify the complex aspects of absorption, distribution, metabolism, and secretion of sesame seed lignans. Once ingested, some lignans are absorbed from the small intestine. In the liver, lignans undergo oxidative biotransformation and demethylation and finally make hydroxylated catechol metabolites. It seems that the main catecholic metabolite of sesame lignan is a compound named heavy (IR,2S,5R,6S)‐6‐(13,4‐dihydroxypheny1)‐2‐(3,4‐methyl lenedioxypheny 1)‐3,7‐dioxabi‐clo be 3,7‐[3,3, O] octane. This compound may be responsible for some of the biological actions of sesame, especially in protecting the liver. Some of them are converted into mammalian lignan, enterolactone (ENL), and to a lesser degree into enterodiol (END), by the intestinal microflora in the proximal part or the upper part of the large intestine. Although ENL and END are animal lignans, and only exist in mammals, they are formed through plant lignans by the enzymatic removal of methyl and hydroxyl groups. ENL and END are absorbed through the hepatic‐intestinal cycle. Also, it is possible that catechol metabolites are secreted into the bile and then metabolized to ENL and END by the intestinal flora of the large intestine. Catechol metabolites eventually form glucuronides and sulfates are secreted into the urine. In addition, there is evidence that part of the metabolism of sesame lignans occurs in the enterocytes of the small intestine before reaching the liver (Scott et al., 1999).

## 4 ATHEROSCLEROSIS

Atherosclerosis is a chronic inflammatory disease resulting from the accumulation of lipids and inflammatory cells in the intima layer of the entire vascular system from the aorta to the coronary arteries and its characteristic is the intimal plaques. The formation of this plaque begins with the deposition of small cholesterol crystals in the intima and the smooth muscle beneath it. Then, the plaques grow and develop the fibrous tissue and surround the smooth muscle, which results in reduction in blood flow (Rafieian‐Kopaei et al., 2014). The production of connective tissue by fibroblasts and calcium deposition in the lesion leads to sclerosis or hardening of the arteries and eventually causes a sudden blockage of blood flow. The rise in lipid and blood sugar is related to the rise in oxidative damage, which affects the antioxidant status and lipoprotein levels (Rafieian‐Kopaei et al., 2014). In addition, obesity, high blood pressure, inflammation, and infectious agents are other causes of atherosclerosis. Studies have shown that plants and phytochemicals with lipid‐lowering effect can prevent atherosclerosis and endothelial damage (Deng et al., 2021; Hou et al., 2020; Loy & Rivlin, 2000; Rahman, 2001).

### 4.1 Sesame and oxidative stress

Various evidences suggest that increased oxidative stress due to the overproduction of free radicals or incompetence of the antioxidant system may develop the atherosclerosis. High concentrations of activated oxygen species can cause membrane peroxidation, protein alteration, DNA failure, activation of neutrophils, disruption of signal transmission pathways, and the regulation of vascular wall cells and heart cells (Kattoor et al., 2017). Low‐density lipoprotein (LDL) cholesterol is not naturally atherogenic but when converted to oxidize LDL form, has the nature of its atherogenicity (Linton et al., 2019). The most important sources that cause oxidative stress in the vessel wall and stimulate the above phenomenon are as follows: activation of nicotine amide adenine dinucleotide phosphate (NADPH), nitric oxide synthase (NOS), myeloperoxidase (MPO), xanthine oxidase, lipoxygenase, and cyclooxygenase (COX; Rabêlo et al., 2010). In addition, increased levels of the antioxidant enzymes glutathione peroxidase (GPx), superoxide dismutase (SOD), and catalase (CAT) are seen; moreover, a decrease in E, A, C vitamins and reduced antioxidant capacity are observed in atherosclerotic patients (Lubrano & Balzan, 2015). MPO is one of the oxidizing enzymes that stimulate the monocytes and neutrophils, and it causes inflammation in the walls of blood vessels to form atherosclerotic plaques and consequently cessation of blood flow to the organs. Another symptom of the disease is a disruption in the production of nitric oxide (NO), as a vasodilator, and the production of reactive oxygen species (ROS), including hydroxyl radicals on the surface of the arteries, which damage the vascular endothelium (Cai & Harrison, 2000; Faruqi, 2013; Ndrepepa et al., 2005; Sukhovershin et al., 2015). The use of antioxidants can be somewhat effective in counteracting oxidative stress. Many studies have shown that boosting the antioxidant system by the consumption of plant sources rich in antioxidants and phenolic compounds can be effective in reducing oxidative stress (Lobo et al., 2010; Table 2).

**TABLE 2:** The potential therapeutic effects of sesame (*Sesamum indicum* L.)

Extract/active compound	Effect	Subject	Dose (μM or μg/mL)	Mechanism	References
Oil (edible oil)	Antioxidative	Human	35 g	Reduction in blood pressure, peroxidation, increase in the activity of enzymatic (CAT, SOD, GPx) and nonenzymatic antioxidants (vitamin C)	Sankar et al. (2006)
Oil	Antioxidative	Rat	4 and 8 mL/kg	Reduction in lipid peroxidation, hydroxyl radicals, and the amount of nitrate, increase in antioxidant enzymes	Hsu and Liu (2004), Hsu et al. (2008)
Oil	Antioxidative	Rat	0.5 mL/kg	Inhibits the expression of renal lipid peroxidation and MPO and reduces ROS	Hsu et al. (2011)
Oil	Antioxidative	Rat	8 mL/kg	Increase in enzymatic antioxidants	Hsu et al. (2005)
Oil	Antioxidative	Rat	200 mL	Increase in enzymatic antioxidants	Ahmad et al. (2006)
Oil	Antioxidative	Rat	5 and 10 mL/kg	Increase in the cardiac endogenous antioxidants	Saleem et al. (2013, 2014)
Oil	Antioxidative	Rat	2% w/w oil supplemented with 6% w/w	Reduction in TBARS, lipid hydro peroxides, blood glucose, increase in GSH and hexokinase	Ramesh et al. (2005)
Oil	Antioxidative	Rat	0.4 g/kg	Decrease in serum glutamate and TBARS	Hemalatha and Raghunath (2004)
Oil	Antioxidative	Rat	10% w/w	Reduction in lipid peroxidation, increase in GSH	Prasanthi and Rajini (2005)
Oil	Antioxidative	Rat	8 mL/kg	Decrease in superoxide anion, hydroxyl radical and lipid peroxidation	Chandrasekaran et al. (2008)
Oil	Antioxidative	Rat	5 mL/kg	Increase in GSH and reduction in TBARS	Abdou et al. (2012)
Oil	Antioxidative	Rat	1.5–3 mL	Protection against EMR, decrease in the cholesterol	Marzook et al. (2014)
Oil	Antioxidative	Rat	1, 2, or 4 mL/kg	Increase in TIMP‐1 and reduction in MMP‐9	Periasamy et al. (2013)
Oil	Antioxidative	Rat	1 mL/kg	Increase in antioxidative enzymes such as GSH, SOD and CAT	Gülcan et al. (2015)
Oil	Antioxidative	Rat	0.5 or 1 mL/kg	Reduction in superoxide anion, hydroxyl radical and lipid peroxidation	Liu and Liu (2017)
Oil	Antioxidative	Rat	1 mL/kg	Reduction in TBARS and GGT	Sotnikova et al. (2009)
Oil	Antioxidative	Rat	1, 2, 4, and 8 mL/kg	Reduction in LPO and DNA	Arumugam and Ramesh (2011)
Oil	Antioxidative	Rat	1, 2, and 4 mL/kg	Reduction in lipid peroxidation, increase in muscular glutathione and GPx	Hsu et al. (2016a, 2016b)
Oil and alpha‐1 lipoic acid	Antioxidative	Rat	5 mL/kg +100 mg/kg	Reduction in AST, ALT, ALP, GGT, cholesterol, TG, LDH, CPK, urea, creatinine	Abdel‐Daim et al. (2016)
Oil	Antioxidative	Mice	20% w/w	Increase in antioxidant enzymes such as SOD, reduce in lipid synthesis enzymes	Woo et al. (2019)
Oil	Antioxidative	Mice	0, 1, 2 and 4 mL/kg	Reduction in serum blood urea nitrogen and creatinine	Li et al. (2012)
Oil	Antioxidative	Mice	4 mL/kg	Increase in TAC	Mosayebi et al. (2007)
Sesamolin	Antioxidative	Rat	1% w/w	Reduction of the TBARS	Kang et al. (1998)
Sesamin	Antioxidative	Rat	2 g/kg	Reduction in TBARS, lipid peroxidation, and hemolysis of red blood cell	Ide et al. (2003)
Sesamin derivative (MMEDA)	Antioxidative	Rat	10 mg/kg	Reduction of oxidative 41% of brain damage, reduction in PGE2	Hung et al. (2017)
Sesamin	Antioxidative	PC12 cells	25, 50, and 100 μmol	Reduction in ROS, NO, and increase in oxidative enzymes, such as SOD, CAT, and GPx	Cao et al. (2013)
Sesamin	Antioxidative	Endothelial cell	12.5–100 μM	Reduction in ox‐LDL, NF‐κB, and increase in SOD‐1	Lee et al. (2009)
3‐ bis (3‐ methoxybenzyl) butane‐1, 4‐diol	Antioxidative	PC12 cells	10 μM	Inhibition of LDH, lipid peroxidation	Hou et al. (2014)
Sesamin and sesamolin	Antioxidative	BV‐2 microglial cells	50–100 μM	Reduction in NO and inhibition of peroxidation	Hou, Chen, et al. (2003), Hou, Huang, et al. (2003)
Sesamin derivative (MMEDA)	Antioxidative	PC12 cells and BV‐2 microglial cells	1, 10, 50 μM	Reduction in ROS, PGs, lipid peroxidation, caspase3 and p‐JNK	Hung et al. (2017)
Sesamin and sesamolin	Antioxidative	PC12 cells	0.5, 5, and 50 μM	Reduction in LDH and inhibition in MAPK and caspase 3	Hou, Chen, et al. (2003), Hou, Huang, et al. (2003)
Seed	Antiinflammatory	Human	40 g	Reduction of hs‐CRP, IL‐6, and MDA	Haghighian et al. (2014)
Oil	Antiinflammatory	Human	1.5 mL	Reduction in pain of knee osteoarthritis	Askari et al. *(* 2019)
Sesamin	Antiinflammatory	Human	200 mg	Reduction in MDA, increase in TAC, and HDL	Helli et al. (2016)
Oil	Antiinflammatory	Rat	1 mL/kg	Reduction in IL‐6 and TNF‐α	Ali et al. (2017)
Oil	Antiinflammatory	Rat	2.5 mg/kg	Reduction in TNF‐α, IL‐6, and IL‐10	Ismail et al. (2018)
Oil	Antiinflammatory	Rat	4 mL/kg	Reduction in fibrosis and acidic mucin	Periasamy et al. (2013)
Oil	Antiinflammatory	Rat	0, 1, 2, or 4 mL/kg	Reduction in TNF‐α, IL‐1β, IL‐4	Hsu et al. (2013)
Oil	Antiinflammatory	Rat	0, 1, 2, or 4 mL/kg	Reduction in IL‐6 and Nrf2	Hsu et al. (2016a, 2016b)
Groundnut oil, rice bran oil Sesame oil	Antiinflammatory	Rat	10% w/t	Reduction in biomarkers of inflammatory and increase in SREBP‐2, PPAR‐γ	Yalagala et al. (2017)
Oil	Antiinflammatory	Rat	20% w/w	Increase in PPAR‐α and CPT‐1	Kim et al. (2017)
Ethanolic extract of seed	Antiinflammatory	Rat	400 and 800 mg/kg	Reduction in IL‐6, inflammation of synovial, cartilage	Ruckmani et al. (2018)
Ethanolic extract of seed	Antiinflammatory	Rat	300 mg/kg	Reduction in inflammatory cytokines	Botelho et al. (2014)
Sesamin	Antiinflammatory	Rat	100 mg/kg	Reduction in blood pressure and LDL cholesterol	Chen et al. (2005)
Sesamin	Antiinflammatory	Rats	15 or 30 mg/kg	Reduction in inflammatory markers	Hsieh et al. (2011)
Sesamin	Antiinflammatory	Rat	10 mg/kg	Reduction in cPLA2, 5‐LOX, BLT‐1, LTC4, TNF‐α, IL‐1β	Yashaswini et al. (2017)
Sesamin	Antiinflammatory	Rat	0–30 mg/kg	Reduction in mucosal lipid peroxidation, NO, TNF‐α, IL‐1β, and MPO	Hsu et al. (2008)
Sesamin	Antiinflammatory	Rat	10 or 20 mg/kg	Reduction in MDA, ROS, caspase 3, α‐synuclein, and increase in SOD	Baluchnejadmojarad et al. (2017)
Oil	Antiinflammatory	Rat	10% w/w	Increase in ca, P, SOD, CAT, GSH, and reduction in MDA, PC, TNF‐α, and CRP	El Wakf et al. (2014)
Sesamin	Antiinflammatory	Rat	0.1% and 1% w/w	Prevention from the production of aortic O^2−^ and endothelial dysfunction	Nakano et al. (2003)
Sesamin	Antiinflammatory	Rat	30 mg/kg	Reduction in TBARS and PC	Khan et al. (2010)
Sesamin	Antiinflammatory	Rat	40, 80, or 160 mg/kg	Reduction in apolipoprotein, ox‐LDL and creatinine and increase in SOD	Zhang et al. (2016)
Sesamin	Antiinflammatory	Rat	5 and 10 mL/kg	Decrease in TBARS and increase in GSH, SOD, CAT	Lv et al. (2015)
Sesamin	Antiinflammatory	Rat	10 mg/kg	Reduction in AST, ALT, CRP, TNF‐α, IL‐1, IL‐6, NO, COX‐2, iNOS	Chiang et al. (2014)
Sesamin	Antiinflammatory	Rat	1 mg/kg	Reduction in lipid peroxidation	Nakai et al. (2003)
Sesamin	Antiinflammatory	Rat	1 or 10 μM	Reduction in reorganization of chondrocytes and increase thickness of cartilage, production of type II collagen, PGs	Phitak et al. (2012)
Aqueous extract of oil	Antiinflammatory	Mice	50 and 250 μg/mL	Increase in TNF‐α, IL‐6, MCP‐1, and VCAM1	Narasimhulu et al. (2018)
Aqueous extract of oil	Antiinflammatory	Mice	0.75 mg	Increase in RCT	Narasimhulu et al. (2018)
Aqueous extract of oil	Antiinflammatory	Mice	10–500 μg/mL	Decrease in IL‐6, TNFα	Selvarajan et al. (2015)
Oil	Antiinflammatory	Mice	5% wt	Prevention from Δ‐5 desaturase activity	Chavali et al. (2001)
Aqueous extract of oil	Antiinflammatory	Mice	340 mg/kg	Increase in metabolism of cholesterol	Narasimhulu et al. (2016)
Ethanol extract of black seeds	Antiinflammatory	Mice	0.5, 1 and 2 mL/kg	Reduction in TNF‐α, IL‐6, NO, MDA	Yang et al. (2018)
Oil and sesamin	Antiinflammatory	Mice	100, 200, 400 mg/kg and 50, 100, 200 mg/kg	Reduction in the synthesis of PGE2	Henriques Monteiro et al. (2014)
Sesamin	Antiinflammatory	Mice	1, 10 and 20 mg/kg	Inhibition of the IL‐4, IL‐5, IL‐13, IgE	Lin et al. (2014)
Sesamin	Antiinflammatory	Mice	10 mg/kg	Increase in p‐JNK	Ma et al. (2014)
Sesamin	Antiinflammatory	Mice	0.5% w/w	Reduction in ICAM‐1	Wu et al. (2010)
Sesamin	Antiinflammatory	Mice	30 and 50 ppm	Increase in 5‐HT, NE, NT3, BDNF, reduction in IBA‐1 and inhibition of the inflammatory cytokines	Zhao et al. (2019)
Seed	Antiinflammatory	Rat	100 and 150 mg/kg	Increase in SOD, improve in AST enzyme	Ibrahim and Al‐feel (2018)
Aqueous extract of oil	Antiinflammatory	MDMs and RAW 264.7 macrophages cells	5 and 25, 50 and 250 μg/mL	Inhibition of the IL‐6, TNF‐α, TLR4 and NF‐kB	Deme et al. (2018)
Aqueous extract of oil	Antiinflammatory	RAW 264.7 macrophages cells	50 and 250 μg/mL	Inhibition of the uptake of Ox‐LDL	Narasimhulu et al. (2018)
Aqueous extract of oil	Antiinflammatory	RAW 264.7 macrophages cells and HUVECS	100 and 200 μg/mL	Reduction in IL‐1α, IL‐6, TNF‐α, MCP‐1, VCAM1, and inhibition of the NF‐kB	Selvarajan et al. (2015)
Ethanolic extract of sesame (*Sesamum indicum* L.) coat	Antiinflammatory	RAW 264.7 macrophages cells	0.01–0.8 mg/mL	Inhibition of the NO, iNOS, NF‐κB and COX‐2	Wang et al. (2007)
Sesamin	Antiinflammatory	PC12 and BV‐2 cells	0.1, 0.5, 1.0, or 2.0 μM	Reduction in ROS, MDA, ERK1/2, P38, caspase 3, COX‐2, and PGE2	Hsieh et al. (2011)
Sesamin derivative (MMEDA)	Antiinflammatory	PC12 and BV‐2 cells	1, 10, and 50 μM	Reduction in ROS, p‐ JNK, caspase 3, and PGE2	Hung et al. (2017)
Sesamin	Antiinflammatory	Primary chondrocytes isolated from 12 osteoarthritic patients.	2.5 and 5 μM	Inhibition of the PGE2, NO, MMP1, 3, and 13, NF‐κB p65, IκBα	Kong et al. (2016)
Sesamin	Antiinflammatory	PC12 cells cultured with N9 microglial cells	1 pM	Reduction in IL‐6, IL‐1β, and TNF‐α	Bournival et al. (2012)
Sesamin	Antiinflammatory	endothelial cell	12.5–100 μM	Reduction in ROS, NF‐κB	Lee et al. (2009)
Sesamin	Antiinflammatory	KBM‐5, A293, H1299, HCT116, and RPMI‐8226	0–50‐μmol/L	Inhibition of the NF‐κB	Harikumar et al. (2010)
Sesamin	Antiinflammatory	HUVECs	10 or 100 μM	Inhibition of the expression of inflammatory factors	Wu et al. (2010)
Sesamin	Antiinflammatory	BV‐2 microglial cell	50 μM	Reduction in TLR4, IL‐1β, IL‐6, TNF‐α, NOS, COX‐2, PGE_2_ and inhibition of the phosphorylation of p‐IkB, p‐p65	Udomruk et al. (2018)
Sesamin	Antiinflammatory	RAW 264.7 macrophage cells	100 μM	Increase in HO‐1	Fukunaga et al. (2014)
Metabolite of sesamin	Antiinflammatory	PC12 cells	10 μM	Increase in HO‐1and ARE/Nrf2	Hamed et al. (2011)
Sesamin	Antiinflammatory	primary chondrocytes isolated from osteoarthritic patients	2.5 and 5 μM	Reduction in MMP1, MMP3, MMP13, p38, and p‐JNK	Phitak et al. (2012)
Ethanol and aqueous leaf extracts	Antiinfection	*E. coli*, *K. pneumoni*, *S. typhii*	100, 200 and 400 mg/mL	Inhibition in the growth of *E. coli*, *K. pneumonia*, *S. typhii*	Ogunsola and Fasola (2014)
Oil	Antiinfection	*S. aureus*	32 mg/mL	Inhibition in the growth of *S. aureus*	Heidari Soureshjani et al. (2017)
Ethanolic, methanolic, and aqueous extracts of leaves	Antiinfection	*S. aureus*, *streptococcus pneumoniae* and *candida albicans*	0.5 mL	Inhibition in the growth of *S. aureus*, *streptococcus pneumoniae*, and *candida albicans*	Bankole et al. (2007)
Oil	Antiinfection	*S. typhii*	10 μL/mL	Inhibition in the growth of *S. typhii*	Saleem (2011)
Silver nanoparticles from extract of sesame	Antiinfection	*E. coli*	200 ppm	Inhibition in the growth of *E. coli*	Bokaeian et al. (2016)
Oil	Antiinfection	*Helicobacter pylori* isolated from patients with chronic gastritis and peptic ulcers	25%, 50%, 75% and 100% w/w	Inhibition in the growth of *helicobacter pylori*	Bakkir and Bakkir (2017)
Seed powder	Antiobesity	Human	50 g	Reduction in weight loss, BMI and waist circumference	Shishehbor et al. (2015)
Methanolic extracts of sesame	Antiobesity	Rat	200 and 400 mg/kg	Reduction in weight of body, glucose, protein, TC, LDL, VLDL, TG	Chinnala et al. (2014)
Oil	Antiobesity	Rat	1.25 mL/kg	Increase in eNOS, NOS	Cebova et al. (2018)
Seed cake	Antiobesity	Rat	2 or 4 g/kg	Reduction in blood glucose, serum cholesterol, serum glucose	Bigoniya et al. (2012)
Oil	Antiobesity	Mice	1% w/w	Reduction in adipose tissue mass, lipid count of serum, and liver, LDL	Pan et al. (2015)
Sesamin	Antidiabetic	Human	200 mg	Reduction in FBS, HbA1c, TNF‐*α*	Mohammad Shahi et al. (2017)
Oil blend	Antidiabetic	Human	35–40 mL	Reduction in FBS, HbA1c, TC, TG, LDL	Devarajan, Chatterjee, Singh et al. (2016), Devarajan, Chatterjee, Urata et al. (2016)
Oil	Antidiabetic	Human	35 g	Reduction in glucose, HbA1c, TC, LDL and TG	Sankar et al. (2011)
Oil	Antidiabetic	Human	900 mL	Reduction in glucose, HbA1c, and increase in insulin	Aslam et al. (2019)
Seed‐based breakfast	Antidiabetic	Human	30 g	Reduction in hs‐CRP	Bahadoran et al. (2015)
Ardeh	Antidiabetic	Human	28 g	Reduction in TG, AIP, TC, LDL	Mirmiran et al. (2013)
Oil+ sesame butter	Antidiabetic	Rat	0.5 g/kg + 1.25 g/kg	Reduction in glucose	Haidari et al. (2016)
Seeds	Antidiabetic	Rat	10% w/w	Reduction in blood glucose	Akanya et al. (2015)
Seeds	Antidiabetic	Rat	5% + 10% w/w	Reduction in FBG, TC, TG, blood urea, nitrogen, creatinine	Ibrahiem (2016)
Lignans	Antidiabetic	Rat	0.25% w/w	Reduction in lipid profile and production of ROS	Dhar et al. (2007)
Sesamin	Antidiabetic	Rat	100 and 200 mg/kg	Reduction in blood pressure and heartbeat	Thuy et al. (2017)
Oil	Antidiabetic	Rat	6% w/w	Reduction in blood glucose, HbA1c, TBARS, lipid hydro peroxides, glucose‐6‐phosphatase, and fructose‐1, 6‐bisphosphatase	Ramesh et al. (2005)
Sesamin	Antidiabetic	Rat	10–20 mg/kg	Increase in NOS and reduction in vascular dysfunction	Baluchnejadmojarad et al. (2013)
Sesamin	Antidiabetic	Mice	100 or 50 mg kg^−1^	Reduction in FBG, glycosylated protein in serum, insulin in serum, TG, cholesterol, FFA, MDA	Hong et al. (2013)
Sesamin	Antidiabetic	Mice	0.2% w/w	Inhibition in blood insulin, blood lipid, superoxide anion, NAD (P)H oxidase	Takada et al. (2015)
Sesamin	Antidiabetic	NIT‐1 pancreatic β‐cells	200 and 400 μg/mL	Reduction in MDA, NO, NOS and iNOS	Lei et al. (2012)
Capsules contain of sesamin	Lipid lowering	Human	3.6 mg	Reduction in the LDL and inhibition in HMGR	Hirata et al. (1996)
White seed	Lipid lowering	Human	40 g	Reduction in TC, LDL, TBARS	Alipoor et al. (2012)
Oil	Lipid lowering	Human	60 g	Reduction in LDL, TG, and increase in HDL	Namayandeh et al. (2013)
Sesamin	Lipid lowering	Rat	0.2% and 0.4% w/w	Inhibition of SREBP‐1 and reduction in lipogenic enzymes	Ide et al. (2001)
Seed powder rich from sesamin and sesamolin	Lipid lowering	Rat	200 g	Reduction in the synthesis of lipids	Sirato‐Yasumoto et al. (2001)
Oil	Lipid lowering	Rat	5% or 10% w/w	Reduction in TG, cholesterol, LDL, VLDL, AST, ALT, GGT, ALP	Taha et al. (2014)
Sesamin	Lipid lowering	Rat	2% w/w	Increase in the metabolism of glucose, cholesterogenesis and lipogenesis of hepatic and oxidation of FA	Ide et al. (2009)
Seed powder	Lipid lowering	Rat	200 g/kg	Reduction in lipogenic enzymes, TG, MDA	Ide et al. (2015)
Sesamin	Lipid lowering	Rat	0.5% w/w	Reduction in cholesterol absorption	Hirose et al. (1991)
Sesamin	Lipid lowering	Rat	5% w/w	Prevention from Δ‐ desaturase activity	Fujiyama‐Fujiwara et al. (1995)
Sesamin	Lipid lowering	Rat	155 μM	Prevention from Δ‐ desaturase activity	Shimizu et al. (1991)
Sesamin	Lipid lowering	Rat	0.5% w/w	Increase in fatty acid oxidation, β‐oxidation of unsaturated fatty acids, mitochondrial and peroxisomal fatty acid oxidation	Ashakumary et al. (1999)
Sesamin	Lipid lowering	Rat	0.5% w/w	Reduction in linoleic acid, α‐linolenic acid, total polyunsaturated fatty acid and increase in dihomo‐γ‐linolenic acid and β‐oxidation of polyunsaturated fatty acid	Mizukuchi et al. (2003)
Sesamin	Lipid lowering	Rat	0.2% w/w	Increase in fatty acid oxidation	Ide et al. (2004)
Sesamin	Lipid lowering	Rat	2 g	Increase in β‐oxidation and reduction in lipogenesis	Kushiro et al. (2004)
Sesamin	Lipid lowering	Rat	0.2% w/w	Increase in fatty acid oxidation	Kushiro et al. (2002)
Sesamin	Lipid lowering	Rat	0.5% w/w	Increase in DGLA and prevention from Δ‐ desaturase activity	Umeda‐Sawada et al. (1995, 1998)
Sesamin	Lipid lowering	Rat	0.2% w/w	Increase in ketone body and reduction in β‐hydroxybutyrate to acetoacetate, TG, lipid secretion	Fukuda et al. (1998)
Sesamin	Lipid lowering	Rat	0.2% w/w	Increase in biliary excretion of cholesterol, ABCG5, ABCG8 and reduction in ApoB	Rogi et al. (2011)
Oil	Lipid lowering	Mice	10% w/w	Reduction in lipid profile, lipid peroxidation, ALP, GR, and increase in antioxidant activity	Korou et al. (2014)
Bugak (pan‐fried unroasted oil)	Lipid lowering	Mice	20 g/100 g of feed	Reduction in TG, TC, LDL, and inhibition of HMGCR and FAS	Kim et al. (2014)
Oil	Lipid lowering	Mice	17% w/w	Increase in ABCA1, ABCA2, Apo E, LCAT, CYP7A1	Narasimhulu et al. (2015)
Sesame lignans	Lipid lowering	Rabbit	50 mg/kg	Reduction in LDL, the expression of platelet‐activating factor acetylhydrolase	Nakamura et al. (2020)
Seed and oil	Lipid lowering	Rabbit	5% or 10% w/w	Reduction in TC, LDL, HDL, SGOT, and SGPT	Asgary et al. (2013)
Seed and oil	Lipid lowering	Rat	2%, 4%, 6% and 8% w/w	Reduction in TC, LDL, HDL, SGOT, and SGPT	Aslam et al. (2020)
Oil	Lipid lowering	primary macrophages isolated from C57/BL6 mice	1–10 μg/mL	Increase in PPARc1, LXRα, and MAPK	Majdalawieh and Ro (2015)
Oil	Antihypertensive	Human	30 mL	Reduction in TG, FBG, HOMA‐IR, MDA, hs‐CRP, TC, and LDL, and improvement in systolic and diastolic blood pressure	Farajbakhsh et al. (2019)
Capsules with sesamin	Antihypertensive	Human	60 mg	Reduction in systolic and diastolic blood pressure	Miyawaki et al. (2009)
Oil (edible oil)	Antihypertensive	Human	35 g	Reduction in blood pressure, TC, LDL, TG, and TBARS	Sankar et al. (2005)
Black sesame meal capsules	Antihypertensive	Human	2.52 g	Reduction in systolic blood pressure, MDA, and increase in vitamin E	Wichitsranoi et al. (2011)
Oil	Antihypertensive	Human	35 g	Improvement in flow‐mediated dilatation and reduction in ICAM	Karatzi et al. (2012, 2013)
Oil blend	Antihypertensive	Human	35–40 mL	Reduction in systolic and diastolic blood pressure, TC, TG, and LDL	Devarajan, Chatterjee, Singh et al. (2016), Devarajan, Chatterjee, Urata et al. (2016)
Sesamin	Antihypertensive	Rat	0.15% w/w	Prevention from cholesterol accumulation	Ogawa et al. (1995)
Sesamin	Antihypertensive	Rat	1000 mg	Reduction in systolic blood pressure, 8‐OHdG and occlusion thrombus of cerebral arterioles	Noguchi et al. (2001)
Sesamin	Antihypertensive	Rat	1% w/w	Reduction in systolic blood pressure, the weight of the left ventricle, and vascular hypertrophy	Matsumura et al. (1995, 1998, 2000), Kita et al. (1995), Nakano et al. (2004)
Sesamin	Antihypertensive	Rat	0.1% w/w	Inhibition in the production of vascular superoxide and reduction in systolic blood pressure	Nakano et al. (2002)
Oil	Antihypertensive	Rat	0.5 or 1 mL/kg	Reduction in the systolic, diastolic blood pressure and abnormalities in ECG	Liu et al. (2014)
Sesamin	Antihypertensive	Rat	>94% purity	Increase in the biosynthesis of NO and reduction in nitrotyrosine, DHFR, superoxide anion	Kong et al. (2015)
Sesame peptide powder	Antihypertensive	Rat	1 and 10 mg/kg	Reduction in systolic blood pressure and suppression in the activity of angiotensin I‐converting enzyme	Nakano, Kwak, et al. (2006), Nakano, Ogura, et al. (2006)
Sesamin	Antihypertensive	Rat	40, 80 and 160 mg/kg	Reduction in p47phox, p22phox, TGF‐β1 and increase in eNOS, MDA	Zhang et al. (2013)
Sesamin	Antihypertensive	Rat	80 and 160 mg/kg	Reduction in TGF‐β1, phosphorylated Smad2, and increase in total antioxidant capacity, SOD	Zhao et al. (2015)
Sesamin	Antihypertensive	Rat	>94% purity	Reduction in MDA, nitro tyrosine, p47phox, and increase in NO	Kong et al. (2009)
Demethylated sesamin metabolites	Antihypertensive	Rat	50 μM	Increase in vasodilation response of endothelium	Nakano, Kwak, et al. (2006), Nakano, Ogura, et al. (2006)
Sesamin	Antihypertensive	Mice	100 mg/kg	Reduction in hypertrophy heart and suppression in fibrosis, inflammation, ROS, phosphorylated ERK1/2, phosphorylated Smad2	Fan et al. (2017)
Sesamin	Antihypertensive	HUVECs	1, 5 and 10 μmoL/L	Increase in NO, eNO, and suppression in ET‐1, ECE‐1	Lee et al. (2005)
Sesamin and sesamolin	Antithrombosis	Mice	(30 mmoL/L:30 mmoL/L) (1 and 10 mmoL/L; 0.1 and 1 mmoL/L)	Antithrombotic effects	Kinugasa et al. (2011)
Sesame or oil	Antithrombosis	Rabbit	1% w/w	Reduction in blood clotting fibrinogen and blood clotting factor VII	Asgary et al. (2011)

Abbreviations: 5‐HT, 5‐hydroxytryptamine; 5‐LOX, *5‐lipoxygenase*; 8‐OHdG, 8‐hydroxy‐2′‐deoxyguanosine; A293, human embryonic kidney carcinoma; ABCA1, ATP‐binding cassette subfamily A member 1; ABCA2, ATP‐binding cassette subfamily A member 2; ABCG5, ATP‐binding cassette subfamily G members 5; ABCG8, ATP‐binding cassette subfamily G members 8; AIP, atherogenic index of plasma; ALP, alkaline phosphatase; ALT, alanine aminotransferase; Apo E, apolipoprotein E; ARE, antioxidant response element; AST, aspartate aminotransferase; BDNF, brain‐derived neurotrophic factor; BLT‐1, block lipid transport‐1; BMI, body mass index; CAT, catalase; COX‐2, cyclooxygenase 2; CPK, creatine phosphokinase; cPLA2, cytosolic phospholipase A2; CPT‐1, carnitine palmitoyl transferase 1; CYP7A1, cytochrome P450 family 7 subfamily A member 1; DGLA, dihomo‐γ‐ linolenic acid; DHFR, dihydrofolate reductase TGF‐β1, transforming growth factor‐β1; *E. coli*, *escherichia coli*; ECE‐1, endothelin‐converting enzyme‐1; ECG, electrocardiography; EMR, electromagnetic radiation; eNOS, endothelial nitric oxide synthase; ERK1/2, extracellular signal‐regulated protein kinases 1 and 2; ET‐1, endothelin 1; FBS, fasting blood sugar; FFA, free fatty acid; GGT, γ‐glutamyltransferase; GPx, glutathione peroxidase; GR, glucocorticoid receptors; GSH, glutathione; H1299, human lung adenocarcinoma; HB, hemoglobin; HbA1c, hemoglobin A1c; Hct, hematocrit; HCT116, human epithelial colon cancer; HDL‐c, high‐density lipoprotein cholesterol; HO‐1, heme oxygenase 1; HOMA‐IR, homeostatic model assessment; hs‐CRP, high‐sensitivity C‐reactive protein; HUVECS, human umbilical vein endothelial cells; IBA‐1, ionized calcium‐binding adaptor molecule 1; ICAM‐1, intracellular adhesion molecule 1; IgE, immunoglobulin E; IL‐10, interleukin10; IL‐13, interleukin 13; IL‐1α, interleukin1α MCP‐1, monocyte chemoattractant protein‐1; IL‐4, interleukin 4; IL‐5, interleukin 5; IL‐6, interleukin 6; iNOS, inducible nitric oxide synthase; IκBα, inhibitor of κB; JNK, c‐Jun N‐terminal kinases; *K. pneumonia*, *klebsiella pneumonia*; KBM‐5, human chronic myeloid leukemia; LCAT, lecithin‐cholesterol acyltransferase; LDH, lactate dehydrogenase; LPO, lipid peroxide; LPS, lipopolysaccharides; LTC4, leukotriene C4; LXRα, liver X receptor alpha; MAPK, mitogen‐activated protein kinases; MDA, malondialdehyde; MMP1, 3 and 13, matrix metallopeptidase1, 3 and 13; MMP‐9, matrix metallopeptidase 9; MPO, myeloperoxidase; NADPH, nicotine amide adenine dinucleotide phosphate; NE, norepinephrine; NF‐κB, nuclear factor kappa‐light‐chain enhancer of activated B cells; NO, nitric oxide; Nrf2, nuclear factor erythroid 2‐related factor 2; NT3, neurotrophin‐3; oxLDL, oxidized low‐density lipoprotein; PC, protein carbonyl; PGE2, prostaglandin E_2;_ PGs, prostaglandins; PPAR‐α, peroxisome proliferator‐activated receptor alpha; PPAR‐γ, peroxisome proliferator‐activated receptor gamma; RBC, red blood cell; RCT, reverse cholesterol transport; ROS, reactive oxygen species; RPMI‐8226, human multiple myeloma; *S. aureus*, *staphylococcus aureus*; *S. typhii*, *salmonella typhi*; SGOT, serum glutamic oxaloacetic transaminase; SGPT, serum glutamic pyruvic transaminase; SOD, superoxide dismutase; SREBP‐1C, sterol regulatory element‐binding protein‐1C; SREBP‐2, sterol regulatory element‐binding protein 2; TAC, total antioxidant capacity; TBARS, thiobarbituric acid reactive substance; TG, three glyceride; TIMP‐1, tissue inhibitors of matrix metalloproteinases 1; TLR4, toll‐like receptor 4; TNF‐α, tumor necrosis factor‐alpha; VCAM1, vascular cell adhesion molecule 1; VLDL, very low‐density lipoprotein; WBC, white blood cell.

#### 4.1.1 Clinical studies

In 18 women and 32 men with hypertension and diabetes, who received diuretics or beta‐blockers, the consumption of sesame oil (edible oil) for 45 days reduced blood pressure, peroxidation, plasma glucose level, glycosylated hemoglobin (HbA1c), total cholesterol (TC), LDL, TG, and the amount of TBARS. However, it raised the activity of enzymatic (CAT, SOD, GPx) and non‐ enzymatic antioxidants (vitamin C; Sankar et al., 2006).

#### 4.1.2 In vivo studies

Treatment of male Wistar rats with sesame oil (8 mL/kg, subcutaneously) was reported to reduce the amount of lipid peroxidation, hydroxyl radicals, and the amount of nitrate induced by lipopolysaccharides (LPS) while raising the activity of antioxidant enzymes, such as SOD and CAT (Hsu & Liu,^2004^). A similar protective effect was seen in rats when the lipid peroxidation induced by cecal ligation and puncture and treated with sesame oil (4 mL/kg, orally) (Hsu et al., 2008). Also, oral treatment of rats with sesame oil (0.5 mL/kg) inhibited the expression of renal lipid peroxidation and MPO and reduced ROS induced by gentamicin‐plus‐iodinated contrast (Hsu et al., 2011). Interestingly, subcutaneous injection of sesame oil (8 mL/kg) raised the activity of enzymatic antioxidants in rats with kidney damage induced by lipopolysaccharide (Hsu et al., 2005). In addition, it has been shown that the consumption of sesame oil (200 mL) increases the activity of enzymatic and non‐enzymatic antioxidants in rats with ischemia induced by occlusion of the right common carotid artery and the right cerebral artery in the midbrain (Ahmad et al., 2006). Also, oral administration of sesame oil (5 and 10 mL/kg) reduced oxidative myocardial damage caused by isoproterenol in rats (Saleem et al., 2014). Similarly, oral administration of sesame oil (5 and 10 mL/kg) raised the cardiac endogenous antioxidants and reduced oxidative stress induced by doxorubicin in rats (Saleem et al., 2013). Furthermore, feeding rats with 2% w/w oil in their diet (contained 6% w/w sesame oil) reduced TBARS, lipid hydroperoxides, and blood glucose, and increased GSH and hexokinase activity in the liver and kidney; it also combats oxidative stress induced by streptozotocin (STZ) (Ramesh et al., 2005). In rats suffering from iron damage, sesame oil (0.4 g/kg) decreased the levels of serum glutamate and TBARS in the liver (Hemalatha & Raghunath, 2004).

Sesame oil (10% w/w)‐containing food supplements reduced lipid peroxidation and raised the amount of GSH against ROS induced by fenvalerate in the liver, brain, thymus, and spleen (Prasanthi & Rajini, 2005).

The use of sesame oil (8 mL/kg) has been shown to decrease the amount of superoxide anion, hydroxyl radical, and lipid peroxidation against ROS induced by acetaminophen in rat liver (Chandrasekaran et al., 2008). Additionally, oral administration of sesame oil (5 mL/kg) increased the level of GSH and reduced the amount of TBARS; also it has a protective effect against oxidative stress induced by cypermethrin in the liver and kidney of rats (Abdou et al., 2012). It has been shown that oral administration of sesame oil (1.5–3 mL) to rats has a protective effect against ROS induced by chronic electromagnetic radiation (EMR) and decreased the cholesterol level in the blood (Marzook et al., 2014). Furthermore, the rise in the expression of tissue inhibitors of matrix metalloproteinase 1 (TIMP‐1) and reduction in the expression of matrix metallopeptidase 9 (MMP‐9) and also the protection against ROS induced by monocrotaline have been shown in rat's colon by 1, 2, or 4 mL/kg dose of sesame oil (Periasamy et al., 2013). In another study, treatment of sesame oil (1 mL/kg, gavage) had protective effects on ROS damage in rats exposed to cyclosporine‐A by increasing the level of antioxidative enzymes, such as GSH, SOD, and CAT in blood, liver, and kidney (Gülcan et al., 2015). It has been reported that treatment of rats with sesame oil (0.5 or 1 mL/kg, gavage) reduced the activity of superoxide anion, hydroxyl radical, and lipid peroxidation in the kidney (Liu & Liu, 2017). Furthermore, sesame oil (1 mL/kg) protected the joint and spleen tissues by reduction in TBARS and γ‐glutamyltransferase (GGT) in rat's plasma (Sotnikova et al., 2017). Oral administration of sesame oil (1, 2, 4, and 8 mL/kg) reduced lipid peroxide (LPO) and DNA damage induced by 4‐nitroquinoline 1‐oxide (4‐NQO) in rat's blood (Arumugam & Ramesh, 2011). Also, feeding with sesame oil (1, 2, and 4 mL/kg) reduced the lipid peroxidation and raised the amount of muscular glutathione and GPx in the model of osteoarthritis in rat (Hsu et al., 2016a, 2016b). Oral administration of sesame oil and α‐1 lipoic acid (5 mL/kg +100 mg/kg) for 4 weeks reduces the level of aspartate aminotransferase (AST), ALT, alkaline phosphatase (ALP), GGT, cholesterol, TG, lactate dehydrogenase (LDH), creatine phosphokinase (CPK), urea, creatinine induced by diazinon (20 mg/kg) in the heart, kidney, and liver of male rats (Abdel‐Daim et al., 2016). Also, in mice suffering from kidney damage induced by high‐lipid diet, sesame oil (20% w/w) increased the antioxidant enzymes such as SOD and reduced the expression of enzymes involved in the synthesis of lipids also; it raised endogenous antioxidants (Woo et al., 2019). Moreover, subcutaneous injection of sesame oil (0, 1, 2 and 4 mL/kg) reduced the level of serum blood urea nitrogen and creatinine in mice model of acute renal damage induced by ferric‐nitrilotriacetate (Fe‐NTA) injection (Li et al., 2012). In one study, intraperitoneal (IP) injection of sesame oil (4 mL/kg) raised the total antioxidant capacity (TAC) in mice (Mosayebi et al., 2007.) Feeding with sesamolin (1% w/w) reduced TBARS caused by ATP‐Fe^3+^/NADPH in the liver microsomes of rats (Kang et al., 1998). Furthermore, feeding of rats with sesamin (2 g/kg) reduced the concentration of TBARS, lipid peroxidation, and hemolysis of red blood cells induced by docosahexaenoic acid (DHA) in rats (Ikeda et al., 2003). IP injection of a derivative of sesamin (1, 2‐bis [(3‐methoxyphenyl) methyl] ethane‐1, 2‐dicarboxylic acid) (MMEDA) (10 mg/kg) for 90 min reduced PGE2 and brain oxidative damage in rat with ischemia induced by occlusion of the right common carotid artery and the right cerebral artery (Hung et al., 2017).

#### 4.1.3 In vitro studies

It has been reported that treatment with sesamin (25, 50, and 100 μmol) reduced the production ROS, NO, and raised the level of oxidative enzymes, such as SOD, CAT, and GPx in PC12 cells (Cao et al., 2013). Also, treatment with sesamin (12.5–100 μM) reduced the oxidized low‐density lipoprotein (oxLDL), NF‐κB and increased SOD‐1 in endothelial cell (Lee et al., 2009). Additionally, treatment with sesamin derivative, 3‐ bis (3‐ methoxybenzyl) butane‐1, 4‐diol (10 μM) protected against the Aβ1‐42‐induced cytotoxicity and apoptosis via multiple mechanisms including released acetylcholine and decreased LDH, released MDA and calcium. The derivative sesame could significantly suppress the c‐Jun N‐terminal kinases (JNK), ERK, p38 MAPK pathways, and Bax expression in PC12 cells exposed to Aβ (Hou et al., 2014). A volume of 50–100 μM of sesamin and sesamolin has an antioxidant activity by reducing NO production and inhibiting the lipid peroxidation in BV‐2 microglial cells induced by LPS (Hou, Chen, et al., 2003; Hou, Huang, et al., 2003). Furthermore, pretreatment with MMEDA (1, 10, 50 μM) reduces ROS, PGs release, lipid peroxidation, the amount of caspase 3, and the active form of JNK in PC12 and BV‐2 microglial cells induced by ischemic and hypoxic neuronal injury (Hung et al., 2017). Similarly, pretreatment with sesamin and sesamolin (0.5, 5, and 50 μM) protected against hypoxia induced by hydrogen peroxide (H_2_O_2_) in PC12 cells via reducing the release of LDH and inhibiting the mitogen‐activated protein kinases (MAPK) pathway and caspase 3 (Hou, Chen, et al., 2003; Hou, Huang, et al., 2003).

**FIGURE 1:**
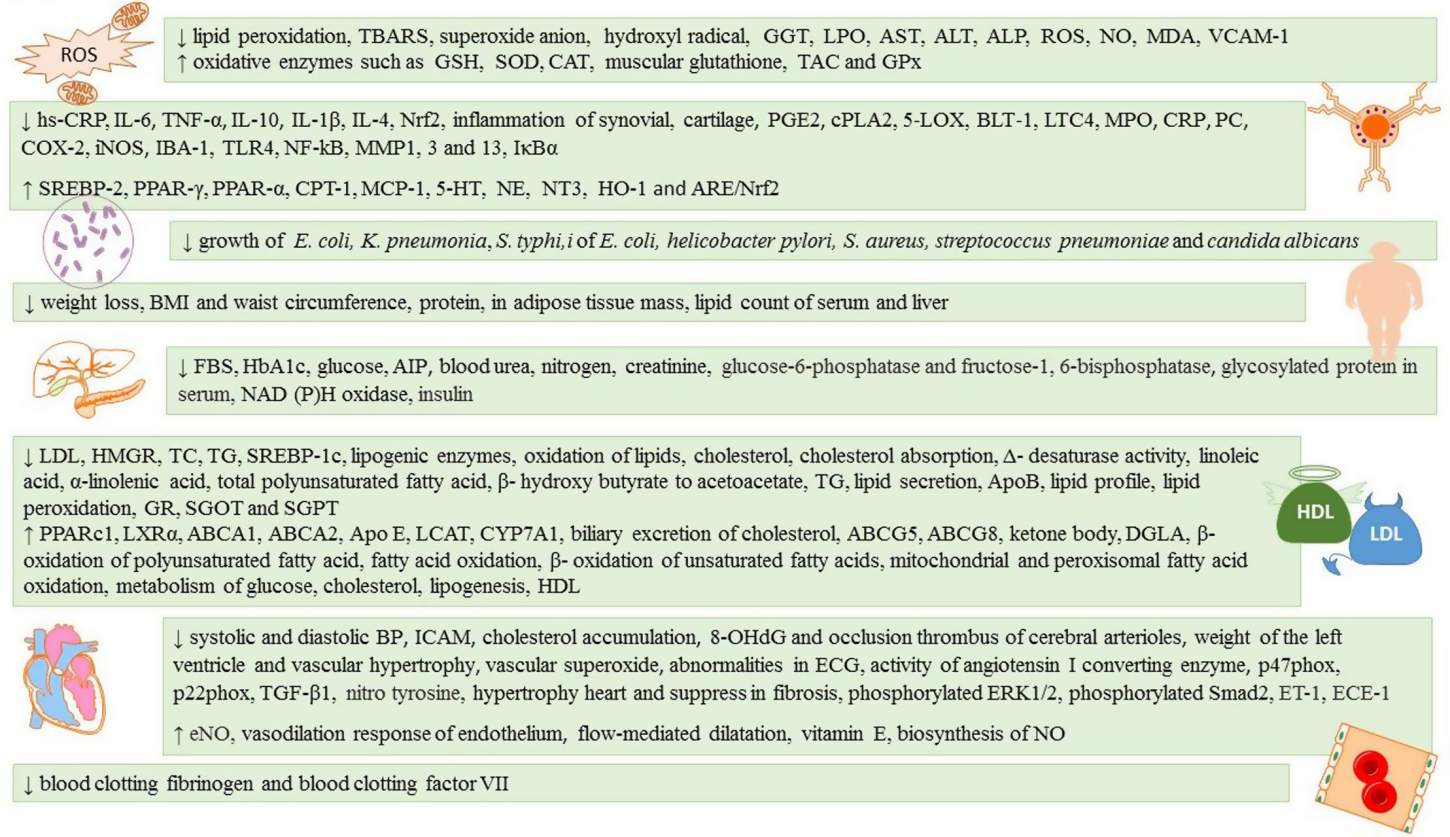
Mechanism of effects of sesame and bioactive compounds (sesamin and sesamolin).

According to the above studies, it can be concluded that sesame seeds reduce ROS by regulating the level of oxidative enzymes, such as SOD, glutathione (GSH), GPx, CAT, and oxidative factors, such as thiobarbituric acid reactive substance (TBARS), malondialdehyde (MDA) sesamin, and sesamolin as the compounds in sesame oil have antioxidative effect by improving the activity of GPx, liver markers (AST ALT, ALP, and GGT), and reducing MDA, lipid peroxidation, and superoxide production (Figure 1).

### 4.2 Sesame and inflammation

The possible connection of endothelial cells with white blood cells, including monocytes and T lymphocytes, happens during vascular inflammation through a molecule attached to the vascular smooth muscle (VASM‐1). White blood cells migrate to the intima of the artery with the help of metalloproteinase and digestion of extracellular matrix that stimulates the production of cytokines. Monocyte and T lymphocytes start to swallow the oxidized lipoproteins, including LDL, and produce foam‐like cells, and their accumulation is associated with the progression of the lesion. Atherosclerotic plaque cells (monocytes, smooth muscle cells, and T cells) secrete IL‐6, complement factor, cytokines, C‐reactive protein (CRP), and NO (Hansson et al., 2006; Libby et al., 1995; Speidl et al., 2005). Macrophages exacerbate the aggregation of plaque by producing interleukin 1 (IL‐1) and tumor necrosis factor‐alpha (TNF‐α). Activated lymphocytes stimulate the proliferation of smooth vascular muscle cells and the production of a dense extracellular substrate, by releasing polypeptide growth factors, which is seen in advanced atherosclerotic lesions (Hansson et al., 2006; Petyaev et al., 2018; Tong et al., 2020; Table 2).

#### 4.2.1 Clinical studies

It has been shown that the consumption of sesame seed (40 g) for 2 months reduces MDA, high‐sensitivity C‐reactive protein (hs‐CRP), and IL‐6 in 50 people with knee osteoarthritis (Haghighian et al., 2014). In addition, it has been reported that the treatment of 104 people' with knee osteoarthritis with sesame oil (1.5 mL) for 4 weeks decreases the pain (Askari et al., 2019). Also, the consumption of sesamin (200 mg/day) could protect against cardiovascular risk factors in patients with rheumatoid arthritis. A decrease in MDA and a rise in TAC and high‐density lipoprotein cholesterol (HDL‐C) levels were reported in the sesamin group. It could decrease systolic blood pressure and lipid profiles (triglycerides, total cholesterol, and low‐density lipoprotein cholesterol (LDL‐C)). The baseline values of body weight and the related factors were also significantly decreased (Helli et al., 2016).

#### 4.2.2 In vivo studies

Oral administration of rats suffering from rheumatoid arthritis, with sesame oil (1 mL/kg) and methotrexate (MTX) (1 and 2 mg/kg) reduced IL‐6, and TNF‐α also increased the amount of IL‐10 (Ali et al., 2017). In another study, in rats suffering from rheumatoid damage induced with injection of complete Freund's adjuvant in the left foot, oral administration of dronabinol (Δ9‐THC)/sesame oil, 2.5 mg/kg for 21 days decreased the deposition of erythrocytes and inflammatory cytokines, including TNF‐α, IL‐6, and IL‐10. Moreover, it dramatically reduced oxidative stress by increasing the activity of antioxidant enzymes, such as CAT, SOD, and GSH (Ismail et al., 2018). In rats suffering from acute colitis, oral administration of sesame oil (4 mL/kg) reduced inflammation, fibrosis, and acidic mucin induced by 2, 4, 6‐tri‐nitro benzene sulfonic acids (TNBS) in rats (Periasamy et al., 2013). Furthermore, oral treatment with sesame oil (0, 1, 2, or 4 mL/kg) reduces TNF‐α, IL‐1β, IL‐4, and acute inflammatory response induced by monosodium urate monohydrate (MSU) crystal in rats (Hsu et al., 2013). In addition, oral treatment with sesame oil (0, 1, 2, or 4 mL/kg) decreased muscular IL‐6, nuclear factor erythroid 2‐related factor 2 (Nrf2) expression, and ROS induced by medial meniscal transection in osteoarthritis rat (Hsu et al., 2016a, 2016b). Adding 10 wt% groundnut oil, rice bran oil, and sesame oil to the diet of male Wistar rats reduced the lipid and inflammation verified by the decrease in cytokines, eicosanoids, NF‐κB, and increase in the expression of sterol regulatory element‐binding protein‐2 (SREBP‐2), PPAR‐γ genes (Yalagala et al., 2017). Feeding of mice with sesame oil (20% w/w) reduced the expression of lipogenic transcription factors and enzymes involved in β‐oxidation of lipids also inhibited the modulation of SREBP‐1 and elevated the expression of peroxisome proliferator‐activated receptor alpha (PPAR‐α) and carnitine palmitoyl transferase 1 (CPT‐1) in hepatic endoplasmic reticulum stress induced by high‐fat diet (Kim et al., 2017). Treatment with ethanol extract of sesame seed (400 and 800 mg/kg) decreased the volume of paw, count of white blood cells, rate of erythrocyte sedimentation, IL‐6, inflammation of synovial, cartilage injury and elevated count of red blood cells, the weight of the body against arthritis induced by Freund's complete adjuvant in rats (Ruckmani et al., 2018). Moreover, IP injection of ethanol extract of sesame seed (300 mg/kg) reduced the amount of inflammatory cytokines against ischemia induced by endothelin‐1 (ET‐1) in rats (Botelho et al., 2014) Oral administration of sesamin (100 mg/kg) reduced ALT, AST, total bilirubin (TBIL), IL‐6, cyclooxygenase −2 (COX‐2), and inhibited NF‐kB elevated by carbon tetrachloride (CCl4) in rats (Chen et al., 2015). Also, consumption of sesamin (15 or 30 mg/kg) decreased markers of inflammation (MDA) and brain injury induced by kainic acid in rats (Hsieh et al., 2011). Oral gavage of sesamin (10 mg/kg) decreases synthesis of cytosolic phospholipase A2 (cPLA2), 5‐lipoxygenase (5‐LOX), block lipid transport‐1(BLT‐1), leukotriene C4 (LTC4), the amount of TNF‐α, monocyte chemoattractant protein‐1(MCP‐1), and IL‐1β against oxidative stress induced by LPS in rats (Yashaswini et al., 2017). In addition, pretreatment of rats with sesamin (10 or 20 mg/kg) decreased the level of MDA, ROS, expression of caspase 3 activity and α‐synuclein proteins, and elevated SOD activity against the formation of brain lesions with 6‐hydroxydopamine (6‐OHDA) (Baluchnejadmojarad et al., 2017). Moreover, feeding with soybean oil (15% w/w) + sesame oil (10% w/w) for 2 months increased the amounts of Ca, P, and the expressions of SOD, CAT, and GSH. However, it reduced the levels of MDA, protein carbonyl (PC), TNF‐α, and CRP against bone loss induced by the deficiency of estrogen hormone in ovariectomized rats (El Wakf et al., 2014). The consumption of sesamin (0.1% and 1% w/w) prevented the increased production of aortic O_2_− and endothelial dysfunction induced by DOCA/salt in hypertensive rats (Nakano et al., 2003). In addition, oral treatment with sesamin (30 mg/kg) reduces TBARS and PC against brain injury induced by reversible middle cerebral artery occlusion in rats (Khan et al., 2010) Intragastric injection of sesamin (40, 80, and 160 mg/kg) reduced the amount of TC, TG, LDL, apolipoprotein, oxLDL, and creatinine and increased the level of SOD against dyslipidemia and kidney injury induced by high‐fat diet in rat (Zhang et al., 2016). IP injection of sesamin (5 and 10 mL/kg) decreased the level of TBARS and increased GSH, SOD, and CAT against oxidative liver injury induced by CCL_4_ in rat (Lv et al., 2015). Gastric gavage of sesamin (10 mg/kg) decreased AST, ALT, CRP, TNF‐α, IL‐1, IL‐6, NO, ROS, p‐JNK, p38, MAPK, COX‐2, inducible nitric oxide synthase (iNOS) and combat the growth arrest and DNA damage in acute hepatic injury induced by LPS/Pb in rat (Chiang et al., 2014). Oral administration of sesamin (1 mg/kg) reduced lipid peroxidation and ROS in rat liver (Nakai et al., 2003). Intra‐articular injection with sesamin (1 or 10 μM) reduced the reorganization of chondrocytes and increased the thickness of cartilage, production of type II collagen, and PGs against *osteoarthritis* induced by papain in the cartilage tissue of rat (Phitak et al., 2012). In addition, it has been shown that the consumption of aqueous extract of sesame oil (50 and 250 μg/mL) has antiinflammatory effects by down‐regulating the expression of inflammatory proteins and reduces TNF‐α, IL‐6, MCP‐1, and vascular cell adhesion molecule 1 (VCAM1) in low‐density lipoprotein receptor knockout (LDLR^−/−^) mice with atherosclerosis induced by high‐fat diet (Narasimhulu et al., 2018). Similarly, the consumption of an aqueous extract of sesame oil (0.75 mg) reduced the expression of inflammatory genes, and increased cholesterol metabolism also reverses cholesterol transport (RCT) in LDLR^−/−^ mice with atherosclerosis induced by high‐fat diet (Narasimhulu et al., 2018). IP injection of an aqueous extract of sesame oil (10–500 μg/mL) reduced inflammatory factors including IL‐6 and TNFα, induced by LPS in mice (Selvarajan et al., 2015). Also, the consumption of sesame oil (5% wt) prevents Δ‐5 desaturase activity and decreased the production of pro‐inflammatory dienoic eicosanoids in cecal ligation and puncture of mice (Chavali et al., 2001). Feeding of (LDLR^−/−^) mice with an aqueous extract of sesame oil (340 mg/kg) regulates the lipid profile via increasing the expression of genes involved in the lipid metabolism and reverses the transport of cholesterol and fight against atherosclerosis induced with high‐fat diet (Narasimhulu et al., 2016). Furthermore, feeding with ethanol extract of black sesame seeds (0.5, 1, and 2 mL/kg) reduced the amount of serum insulin, TNF‐α, IL‐6, NO, MDA, glucose, and lipid accumulation in the liver. Also, it attenuated the expression of p‐JNK1/2/3, phospho‐NF‐κB p65, phospho‐insulin receptor substrate 1 (IRS1), phospho‐IKK α/β, and elevated the level of x‐box‐binding protein 1 (XBP1), GSH, vitamin C, Nrf2, SOD, CAT, and GPx in nonalcoholic fatty liver disease in fructose‐fed mice (Yang et al., 2018). In mice suffering from Paw edema, oral administration with sesame oil (100, 200, and 400 mg/kg) and sesamin (50, 100, and 200 mg/kg) prevented the synthesis of PGE2, production of pleural exudate, and migration of leucocyte (Henriques Monteiro et al., 2014). IP injection of sesamin (1, 10 and 20 mg/kg) prevented the expression of interleukin 4 (IL‐4), interleukin 5 (IL‐5), interleukin 13 (IL‐13), serum immunoglobulin E (IgE), and reduced the number of eosinophils, other inflammatory cells against allergen and airway inflammation induced by ovalbumin (OVA) in asthmatic mice (Lin et al., 2014). In addition, oral treatment with sesamin (10 mg/kg) increased the level of p‐JNK against oxidative stress induced by CCl4 in mice (Ma et al., 2014). Consumption of sesamin (0.5% w/w) reduced the expression of intracellular adhesion molecule 1(ICAM‐1) and intima thickness in atherosclerosis induced by high‐fat diets in apolipoprotein E (apoE)‐deficient mice (Wu et al., 2010). In other study, oral treatment with sesamin (30 and 50 ppm) increases the amount of 5‐hydroxytryptamine (5‐HT), norepinephrine (NE), neurotrophin‐3 (NT3), brain‐derived neurotrophic factor (BDNF), and improved depression. In addition, it reduced the expression of ionized calcium‐binding adaptor molecule 1 (IBA‐1) and suppressed the generation of inflammatory cytokines (iNOS, COX‐2, TNF‐α, and IL‐1β) against depression induced by chronic unpredictable mild stress in mice (Zhao et al., 2019). Treatment with raw, roasted, and fermented sesame seeds (100 and 150 mg/kg diet) improved AST enzyme and increased the activity of oxidative enzymes including SOD in atherosclerosis induced by atherogenic diet in rats. Furthermore, it improved the activity of the pedal against inflammation induced by 0.1 mL formalin (4%) in rats (Ibrahim et al., 2020).

#### 4.2.3 In vitro studies

Pretreatment with aqueous extract of sesame oil (5, 25, 50, and 250 μg/mL for 2 h) has antiinflammatory and antiatherosclerotic properties against inflammation induced by LPS via preventing of IL‐6, TNF‐α, TLR4, and NF‐kB in monocyte‐derived macrophages (MDMs) and RAW 264.7 macrophages cells (Deme et al. 2018). Similarly, pretreatment with an aqueous extract of sesame oil (50 and 250 μg/mL) inhibited the uptake of Ox‐LDL in RAW 264.7 mouse macrophages against atherosclerosis induced by Ox‐LDL (Narasimhulu et al., 2018). Treatment with aqueous extract of sesame oil (100 and 200 μg/mL) reduced IL‐1α, IL‐6, TNF‐α, MCP‐1, VCAM1, and inhibited NF‐kB induced by LPS in RAW 264.7 mouse macrophages and human umbilical vein endothelial cells (HUVECS; Selvarajan et al., 2015). Also, pretreatment with ethanol extract of sesame coat (0.01–0.8 mg/mL) elevated the amount of GSH, GPx, GSH reductase (GR), GSH transferase (GSTs), CAT, and suppressed the generation of NO, iNOS, NF‐κB, and COX‐2 against the production of inflammatory mediators induced by LPS in RAW 264.7 macrophages (Wang et al., 2007). Pretreatment with sesamin (0.1, 0.5, 1.0, or 2.0 μM) reduced ROS, MDA, extracellular signal‐regulated protein kinases 1 and 2 (ERK1/2), P38, caspase 3, COX‐2, and PGE2 induced by kainic acid in PC12 and BV‐2 cells (Hsieh et al., 2011). Furthermore, treatment with sesamin (2.5 and 5 μM) inhibited the generation of PGE2, NO, matrix metallopeptidase 1, 3, and 13 (MMP1, 3 and 13), NF‐κB p65, and inhibitor of κB (IκBα) induced by IL‐1β in primary chondrocytes isolated from 12 osteoarthritic patients. In addition, it increased the expression of Nrf2 and heme oxygenase 1(HO‐1) (Kong et al., 2016). Pretreatment with sesamin (1 pm) reduced the expiration of IL‐6, IL‐1β, and TNF‐α proteins in PC12 cells cultured with N9 microglial cells (Bournival et al., 2012). Similarly, pretreatment with sesamin (12.5–100 μM) reduced the production of ROS, NF‐κB, and activated SOD1 against inflammation and oxidative stress induced by ox‐LDL in endothelial cells (Lee et al., 2009). Similarly, pretreatment with sesamin (0–50‐μmol/L) suppressed the pathway linked to the NF‐κB signaling against chronic diseases induced by TNF in KBM‐5 (human chronic myeloid leukemia), A293 (human embryonic kidney carcinoma), H1299 (human lung adenocarcinoma), HCT116 (human epithelial colon cancer), and RPMI‐8226 (human multiple myeloma; Harikumar et al., 2010). During inflammation, TNF‐α increases human antigen R (HuR) translocation and its binding to 3′UTR of ICAM‐1 mRNA which boosts the adhesion of white blood and aortic endothelial cells and eventually inflammation and atherosclerosis. Sesamin reduced the expression of ICAM‐1 gene both exogenously and endogenously, so it attenuated the adhesion of white blood cells to aortic endothelial cells. It has been shown that pretreatment of HUVECs with sesamin (10 or 100 μM) inhibited the expression of inflammatory factors: NF‐kB p65 and CAM‐1 also decreased the cell adhesion via down‐regulating the ERK12 and p38 (Wu et al., 2010). Pretreatment with sesamin (50 μM) reduces the expression of toll‐like receptor 4 (TLR4), IL‐1β, IL‐6, TNF‐α, NOS, the amount of COX‐2, generation of PGE_2_ and inhibits the phosphorylation of p‐IkB, p‐p65. Additionally, it decreased the phosphorylation of JNK and p38 against neurotoxicity induced by LPS in BV‐2 microglial cell (Udomruk et al., 2018). The study reported that pretreatment with sesamin (100 μM) increases the amount of HO‐1 protein and ceases the degradation of HO‐1 protein via suppressing its ubiquitination against inflammation induced by LPS in RAW 264.7 macrophage cells (Fukunaga et al., 2014). Similarly, pretreatment with the metabolite of sesamin (10 μM) increased the amount of HO‐1 protein and activated the antioxidant response element (ARE/Nrf2) against oxidative stress induced by H_2_O_2_ in PC12 cells (Hamed et al., 2011). Pretreatment with sesamin (2.5 and 5 μM) reduced the expression of MMP1, MMP3, MMP13, p38, and JNK against inflammation induced by IL‐1β in primary chondrocytes isolated from osteoarthritic patients (Phitak et al., 2012). Moreover, pretreatment with sesame extract (1–100 μg/mL) increased the secretion of Th1 cytokine, inhibits the secretion of Th2 cytokine, IL‐6, TNFα, and NO, and elevated the proliferation of splenocytes and function of primary macrophages isolated from BALB/c and C57BL/6 mice (Majdalawieh et al., 2020).

According to the studies reviewed above, it seems that antiinflammatory effects of sesame oil are attributed to the reduction in pre‐inflammatory cytokines including interleukin 1α (IL‐1α), interleukin 1β (IL‐1β), IL‐6, and TNF‐α. Studies show that sesame oil and lignans, including sesamol and sesamolin, have antiinflammatory effects by inhibiting COX‐2. It has been shown that sesamin triggers the accumulation of dihomo‐γ‐ linolenic acid (DGLA) by inhibiting the activity of Δ5‐desaturase and prevents the conversion of DGLA to arachidonic acid in the cell membrane. Eventually, it reduces the production of inflammatory factors (Bankole et al., 2007). In addition, modulation of NF‐κB pathway by sesamin, which is initiated by inflammatory agents, has been addressed. When the activity of beta‐activated kinase 1 (TAK1) protein is inhibited, it decreases the activity of NF‐κB via inhibition of the phosphorylation of inhibitor of nuclear factor‐κB (IκB) kinase (IKK) (Harikumar et al., 2010; Figure 1).

### 4.3 Sesame and infection

Infection is one of the most important issues in the occurrence of atherosclerosis. The growth of bacteria or viruses in vascular cells stimulates the secretion of cytokines and CRP, which are promoters of atherosclerosis. *Chlamydia pneumonia*, *Porphyromonas gingivalis*, *Helicobacter pylori*, influenza A virus, hepatitis C, cytomegalovirus, and human immunodeficiency virus have been identified as inducers which increased the risk of cardiovascular disease (Campbell & Rosenfeld, 2015). Ethanol and aqueous leaf extracts of sesame (100, 200, and 400 mg/mL) suppressed the growth of *Escherichia coli*, *Klebsiella pneumonia*, and *Salmonella typhi* (Ogunsola & Fasola, 2014). Furthermore, it has been shown that sesame oil (32 mg/mL) suppresses the growth of *Staphylococcus aureus* (*S. aureus*) (Heidari Soureshjani et al., 2017). Also, ethanol, methanol, and aqueous extracts of sesame leave have inhibitory effects against *S. aureus*, *Streptococcus pneumoniae*, and *Candida albicans* (Bankole et al., 2007). In addition, sesame oil (10 μL/mL) was potently combative against *S. typhii* (Saleem, 2011). In another study, it was revealed that synthesized silver nanoparticles containing the extract of sesame (200 ppm) combat five types of isolates of *E. coli* (Bokaeian et al., 2016). Sesame oil (25%, 50%, 75%, and 100% ww) has an inhibitory effect against *Helicobacter pylori* isolated from patients with chronic gastritis and peptic ulcers (Bakkir & Bakkir, 2017; Table 2).

### 4.4 Sesame and obesity

Obesity increases the risk factors for heart disease including high blood pressure, cholesterol abnormalities, and type 2 diabetes. Additionally, being overweight increases the risk of metabolic syndrome (a group of risk factors for heart disease including high blood pressure, low HDL cholesterol, high TG, high blood glucose, and high waist size). In addition, high blood pressure from obesity accelerated the plaque formation in the blood vessels, making them prone to rupture. In addition, latent inflammation caused by obesity in the body increases the risk of atherosclerosis and plaque hardening on the walls of arteries. Obesity also causes the release of inflammatory factors in the blood that may rupture the vascular plaque and causes heart attack. In addition, abnormalities in the metabolism of lipid, vascular endothelial function, and adipocytokines balancing also insulin resistance, have been linked to the prevalence of obesity and atherosclerosis (Lovren et al., 2015) Table 2.

#### 4.4.1 Clinical studies

It has been shown that the consumption of sesame seed powder (50 g) for 6 weeks reduced weight loss, BMI, and waist circumference in 46 women with metabolic syndrome (Shishehbor et al., 2015).

#### 4.4.2 In vivo studies

Feeding with methanol extracts of sesame (200 and 400 mg/kg) reduced the body weight, the amount of glucose, protein, TC, LDL, very low‐density lipoprotein (VLDL), TG, and elevated amount of HDL in rat fed high‐fat diet (Chinnala et al., 2014). Also, co‐treatment with sesame oil (1.25 mL/kg) and simvastatin (15 mg/kg) elevated the expression of phosphorylated endothelial nitric oxide synthase (eNOS) protein, NOS activity and reduced the oxidative load in obese Zucker rats (Cebova et al., 2018). Furthermore, the consumption of sesame seed cake (2 or 4 g/kg) decreases the level of blood glucose, serum cholesterol, serum glucose, and prompted the glucose tolerance in rat fed by high‐fructose diet (Bigoniya et al., 2012). Besides, sesame oil (1% w/w) reduced adipose tissue mass, lipid count of serum and liver, LDL, and inhibited the expression of lipogenic enzymes including: PPAR‐γ, sterol regulatory element‐binding protein‐1C (SREBP‐1C), stearoyl‐CoA desaturase 1(SCD‐1), Fas, acetyl‐CoA carboxylase (ACC), lysinuric protein intolerance (LPI), and malic enzyme in C57BL/6 mice fed with high‐fat diet (Pan et al., 2015).

Based on the above studies, consumption of sesame seeds and their products seems to play a significant role in weight control. In addition, sesame lignans may cause weight loss due to the fat‐burning effect of the oil, and increase in the expression of uncoupling proteins in the inner mitochondrial membrane. These proteins provide the energy needed for oxidative phosphorylation. Also, lignans in sesame increase the expression of the enzymes involved in β‐oxidation of lipids and increase the cellular capacity for fat burning (Kushiro et al., 2004, 2002; Figure 1).

### 4.5 Sesame and diabetes

People with diabetes are two to six times more likely to develop atherosclerosis than non‐diabetics. The process of accelerating atherosclerosis in person with *diabetes* can be attributed to the decrease in the production of NO. NO, which is normally secreted from vascular endothelial cells, causes vasodilation and proper blood flow inside the arteries. On the other hand, NO protects the reaction of platelets and leukocytes with the walls of blood vessels and is followed by intravascular damage. In diabetic patients, the level of NO is decreased, as a result, disruption in the vasodilation process increases the platelet aggregation (Hamed et al., 2011). Moreover, in diabetic patients, the entire coagulation cascade is impaired. Disruption in the functions of insulin as a natural platelet antagonist and inflammatory responses due to fluctuations in blood sugar level in diabetic patients accelerate the platelet adhesion to the blood vessels (Barlovic et al., 2011; Duarte et al., 2019; Ko et al., 2018, 2019). Inflammatory responses characterized by elevated CRP cause vascular disease in patients (Zhang et al., 2014). Furthermore, endothelin‐1 (responsible for the dysfunction of endothelium and secreted from endothelial cells, vascular wall smooth muscle (Kalani, 2008) and inflammatory cells) is secreted more in diabetic patients than in healthy individuals (Gogg et al., 2009; Table 2).

#### 4.5.1 Clinical studies

In 48 patients with type 2 diabetes, the consumption of sesamin (200 mg) for 8 weeks reduced the amount of FBS, HbA1c, TNF‐α, waist circumference, hip circumference, body adiposity index (BAI), IL‐6, and elevates the amount of adiponectin (Mohammad Shahi et al., 2017). Moreover, it has been shown that treatment with sesame oil blend (35–40 mL) and glibenclamide (5 mg) for 8 weeks reduces the amount of FBS, HbA1c, TC, TG, LDL, and increases HDL in 300 patients with type 2 diabetes (Devarajan, Chatterjee, Singh, et al., 2016; Devarajan, Chatterjee, Urata, et al., 2016). Besides, the consumption of sesame oil (35 g) and glibenclamide (5 mg) for 60 days improved antidiabetic effects by reduction in the level of glucose, HbA1c, TC, LDL, and TG and elevation of the level of HDL in 33 patients with type 2 diabetes (Sankar et al., 2011). In another study, in 46 patients suffering from type 2 diabetes, the consumption of sesame oil (900 mL) decreased glucose, HbA1c and increases insulin, the expression of SOD, CAT, and GPx (Aslam et al., 2019). Furthermore, the consumption of sesame seed‐based breakfast (Tahini 30 g) for 2 weeks reduced the level of hs‐CRP against inflammation induced by type 2 diabetes in 41 patients (Bahadoran et al., 2015). Consumption of 28 g Ardeh (sesame paste) for 6 weeks reduced the level of TG, atherogenic index of plasma (AIP), TC, LDL, and increased the level of HDL in 41 persons with type 2 diabetes (Mirmiran et al., 2013).

#### 4.5.2 In vivo studies

Oral treatment with sesame oil (0.5 g/kg) and sesame butter (1.25 g/kg) for 6 weeks decreased the level of glucose and elevated the level of HD against diabetic induced by STZ in rat (Haidari et al., 2016). Also, feeding with sesame diet (10% w/w) reduced the level of blood glucose, cholesterol, TG, LDL, and increased HDL in alloxan‐diabetic rats (Akanya et al., 2015).

Similarly, diet supplementation with *Nigella sativa* (5% + 10% w/w) and sesame seeds (5% + 10% w/w) reduced lymphocytes count and generation of TNF‐α, IL4, IL8, the level of FBG, TC, TG, blood urea nitrogen, and creatinine; however, it increased the expression of SOD, GPx, and CAT in alloxan‐induced diabetic rats (Ibrahiem, 2016). In addition, the consumption of sesame lignans and tocopherols (0.25% w/w sesame lignin+0.25% w/w α tocopherol) decreased lipid profile and production of ROS in diabetic (DM) rat (Dhar et al., 2007). In rats suffering from cardiac dysfunction caused by type 1 diabetes with STZ, oral treatment with sesamin (100 and 200 mg/kg) reduced blood pressure and heartbeat (Thuy et al., 2017). Furthermore, consumption of sesame oil (6% w/w) reduced the amount of blood glucose, HbA1c, TBARS, lipid hydro peroxides, the expression of glucose‐6‐phosphatase, and fructose‐1, 6‐bisphosphatase. However, it increased the level of hemoglobin, vitamin E, GSH, and expression of hexokinase against diabetes induced by STZ in rat (Ramesh et al., 2005). Sesamin (10–20 mg/kg, gavage) attenuated the contractile response to phenylephrine and elevated the relaxation response to acetylcholine in endothelium‐intact aortic rings model of vascular dysfunction through a rise in NOS in STZ‐induced diabetic rat (Baluchnejadmojarad et al., 2013). Also, feeding mice with sesamin (0.2% w/w) for 8 weeks inhibited the elevation in the amount of blood insulin, lipid, superoxide anion, and the expression of NAD (P) H oxidase induced by high‐fat diet. In addition, it increased the capacity of exercise and the expression of citrate synthase in the skeletal muscle of diabetic mice (Takada et al., 2015). Additionally, treatment with sesamin (100 or 50 mg/kg) for 2 weeks reduced the amount of FBG, glycosylated protein in serum, insulin in serum, TG, cholesterol, FFA, MDA, and increased the ability of insulin to bind to its receptors on the liver membrane and the amount of glycogen in the liver. Moreover, it improved the histopathological changes of pancreas and the expression of GSH, SOD, GPx against hyperglycemia, hyperlipidemia, and insulin resistance in KK‐Ay mice with type 2 diabetes (Hong et al., 2013).

#### 4.5.3 In vitro studies

Pretreatment with sesamin (200 and 400 μg/mL for 24 h) reduced the level of MDA, generation of NO and the expression of NOS and iNOS against the damage induced by STZ in NIT‐1 pancreatic β‐cells (Lei et al., 2012).

According to the studies conducted above, it seems that sesame seeds have antidiabetic effects by reducing the FBS, glucose, AIP, glycosylated protein, and insulin and increasing the amount of glycogen in the liver. Also, as mentioned, consumption of sesame seeds reduces inflammatory factors such as hs‐CRP, TNF‐α, IL4, and IL8 in diabetic patients. Therefore, it can be concluded that sesame seeds have shown favorable effects through the reduction in special parameters, especially the reduction in inflammatory factors, which are the causes of dysfunction in the endothelium of the blood vessels of diabetics and may predispose to atherosclerosis (Figure 1).

### 4.6 Sesame and lipid profile

Hyperlipidemia is one of the known risk factors of the coronary artery disease and atherosclerosis (Smith Jr et al., 2004). High LDL is directly correlated to coronary artery disease, HDL is one of the strongest protective factors against atherosclerosis (Rudel & Kesäniemi, 2000) and a slight increase in TG prompts the risk of coronary heart disease and formation of new lesions (Assmann & Schulte, 1992; Bjørndal et al., 2020; Hokanson & Austin, 1996; Table 2).

#### 4.6.1 Clinical studies

It has been reported that treatment with sesamin (3.6 mg) + vitamin E (180 mg) capsules for 4 and 8 weeks reduces the level of cholesterol in serum (LDL) and inhibits HMG‐CoA reductase (HMGR) in 20 males with hypercholesterolemia (Hirata et al., 1996). In 38 patients suffering from hyperlipidemia, treatment with white sesame seed (40 g) for 60 days reduced the level of TC, LDL, TBARS, and increased the gene expression of GPX and SAD (Alipoor et al., 2012). Moreover, consumption of sesame oil (60 g) for 1 month reduced the level of LDL, TG, and increased the level of HDL. Also, it reduced body weight and waist circumference in 48 patients with hypercholesterolemia (Namayandeh et al., 2013).

#### 4.6.2 In vivo studies

It has been shown that feeding rats with sesamin (0.2% and 0.4% w/w) for 15 days inhibits the expression of SREBP‐1 and reduces the gene expression of ACC, fatty acid synthase, ATP‐citrate lyase (ACLY), and glucose‐6‐phosphate dehydrogenase (G6PD) involved in the lipogenesis (Ide et al., 2001). Also, feeding male rats with sesame seed powder which is rich in sesamin and sesamolin (200 g/kg) increased the amount of fatty acid oxidation of hepatic mitochondria. In addition, it reduced the activity of enzymes involved in the synthesis of fatty acids and TG (Sirato‐Yasumoto et al., 2001). Similarly, elevation in the enzymes involved in the oxidation of fatty acid in rat liver seen when sesamin (0.2% w/w) plus fish oil (8% w/w; Ide et al., 2004) or sesamin (0.2% w/w) alone was fed for 15 days in rats (Kushiro et al., 2002). It has been reported that the consumption of sesame oil (5% or 10% w/w) reduced lipid profiles in serum and liver including: TG, cholesterol, LDL, VLDL, and decreased the expression of liver enzymes including: AST, ALT, GGT, ALP. Also, it is increased in HDL, adiponectin, and thyroid hormones against the hyperlipidemia induced by triton WR1339 in rat (Taha et al., 2014). Moreover, feeding with sesamin (2% w/w) for 15 days elevated the gene expression of enzymes involved in metabolism of glucose, cholesterogenesis, and lipogenesis in the liver. In addition, it stimulated the oxidation of fatty acid in the liver of rat (Ide et al., 2009). Moreover, sesame seed powder (200 g/kg) decreased the gene expression of lipogenic enzymes and the level of TG and MDA in rat (Ide et al., 2015). In addition, supplementing with sesamin (0.5% w/w) for 4 weeks decreased cholesterol absorption through the lymph and increased its extraction in the feces of rat (Hirose et al., 1991). Besides, feeding with sesamin (5% w/w) for 4 weeks prevented the Δ‐desaturation of *n*‐6 fatty acids in rat hepatocytes (Fujiyama‐Fujiwara et al., 1995). Similarly, it has been shown that treatment with sesamin (155 μM) for 4 week inhibits Δ5 desaturase and the biosynthesis PUFA in rat liver microsomes (Shimizu et al., 1991). Consumption of sesamin (0.5% w/w) for 15 days elevated the genes expression of enzymes involved in the β‐oxidation of unsaturated fatty acids and mitochondrial and peroxisomal oxidation of fatty acid also reduced the expression of lipogenic enzyme in rat liver (Ashakumary et al., 1999). Feeding with sesamin (0.5% w/w) for 4 weeks decreased the level concentration of linoleic acid, α‐linolenic acid, and total PUFA and elevated the level of dihomo‐γ‐linolenic acid, and the gene expression of enzyme involved in the β‐oxidation of PPUFA in high‐fat diet rats (Mizukuchi et al., 2003). In addition, sesamin (2 g/kg for 15 days) elevated the gene expression of enzymes involved in the β‐oxidation and reduced lipogenesis in rat (Kushiro et al., 2004). Feeding with sesamin (0.5% w/w) increased the amount of DGLA and prevented the Δ‐desaturation of *n*‐6 fatty acids in rat liver microsomes (Umeda‐Sawada et al., 1995, 1998). Furthermore, sesamin (0.2% w/w) for 16 days stimulated the production of ketone body and reduced TG, lipid secretion. Also, feeding sesamin lowered the ratio of β‐hydroxyl butyrate to acetoacetate in rat liver (Fukuda et al., 1998). Moreover, feeding with sesamin (0.2% w/w) and *α*‐tocopherol (1% w/w) for 10 days fought against high‐cholesterol diet in rats (Rogi et al., 2011). In one study, supplementing with sesame oil (10% w/w) or N‐acetylcysteine (NAC) (230 mg/kg) reduced the level of lipid profile, lipid peroxidation, ALP, and hypothalamic glucocorticoid receptors (GR) and prevented the hepatic damage in mice fed with high‐cholesterol‐enriched diet (Korou et al., 2014). It has been shown that bugak (pan‐fried unroasted sesame oil) (20 g/100 g of feeding diet) reduces TG, TC, and LDL, and inhibits the expression of HMGCR and hepatic FAS in LDLR^−/−^ mice (Kim et al., 2014). Similarly, feeding LDLR^−/−^ mice with sesame oil (17% w/w) increased the expression of genes involved in the metabolism of cholesterol and reversed the cholesterol transfer by LDL in the liver. Increase in ATP‐binding cassette subfamily A member 1 (ABCA1), ATP‐binding cassette subfamily A member 2 (ABCA2), apolipoprotein E (Apo E), lecithin‐cholesterol acyltransferase (LCAT), cytochrome P450 family 7 subfamilies A member 1(CYP7A1) fight against atherosclerosis induced by consumption of high‐fat diet (Narasimhulu et al., 2015). Also, sesame lignans (50 mg/kg, orally) reduced the expression of platelet‐activating factor acetylhydrolase and prolonged the LDL oxidation delay time in rabbits fed with fat/cholesterol‐enriched diet (Nakamura et al., 2020).

Moreover, sesame seed (5% or 10% w/w) or sesame oil (2%, 4%, 6%, and 8% w/w) lowered the level of TC, LDL, HDL, serum glutamic oxaloacetic transaminase (SGOT), and serum glutamic pyruvic transaminase (SGPT) hypercholesterolemic rabbits and rats fed with 1% w/w cholesterol (Asgary et al., 2013; Aslam et al., 2020).

#### 4.6.3 In vitro studies

It has been shown that pretreatment with sesame oil (1–10 μg/mL) increases the gene expression of PPARc1, liver X receptor alpha (LXRα), and MAPK. Also, it increases the cholesterol efflux in primary macrophages isolated from C57/BL6 mice (Majdalawieh & Ro, 2015).

The use of sesame oil in Asian cultures has inspired many studies on its beneficial effects on lipid profile. For example, in China, 28 kg of edible oil, especially sesame oil, is used annually. Sesamin appears to induce lipid oxidation in the liver by activating PPAR. Also, it reduces the gene expression of hepatic lipogenic enzyme with down‐regulation of SREBP‐1 transcription factor (Ide et al., 2003; Majdalawieh et al., 2020). In addition, it prevented the Δ‐desaturation of *n*‐6 fatty acids, inhibited the expression of HMGCR and hepatic FAS, and reduced the amount of plasma cholesterol, TG, and LDL. The proposed mechanism is an increase in the biliary excretion of cholesterol in the liver via increasing in gene expression of ATP‐binding cassette subfamily G members 5 (ABCG5), ATP‐binding cassette subfamily G members 8 (ABCG8) and a reduction in the secretion of apolipoprotein B (ApoB) via decreasing in the gene expression of apolipoprotein A4 (ApoA4) (Rogi et al., 2011). Therefore, it seems that sesame seeds combated atherosclerosis induced by the consumption of high‐fat diet through the mentioned possible mechanisms (Figure 1). Also, β‐carbonyls such as Harman and nor‐Harman have been detected in sesame oil (Liu et al., 2022). Perhaps, the existence of these active compounds is a source of the effects of sesame oil in dealing with dyslipidemia. Of course, investigating this issue requires more research.

### 4.7 Sesame and hypertension

Hypertensive vascular disease affects large and small arteries and arterioles and is characterized by the thickening of the fibromuscular layer of the intima and media and finally narrowing of arteries and arterioles. The physical pressure of hypertension on the arterial wall also leads to the exacerbation and acceleration of atherosclerosis, especially in coronary arteries. In addition, high blood pressure appears to increase the susceptibility of small and large arteries to atherosclerosis. Therefore, a patient with high blood pressure is a candidate for high blood pressure and atherosclerotic diseases, which leads to blockage of large and small arteries, resulting in myocardial infarction (Hollander, 1976) (Table 2).

#### 4.7.1 Clinical studies

It has been reported that in 25 patients suffering from metabolic syndrome, co‐consumption of sesame oil (30 mL) and vitamin E (400 mg) reduces the level of TG, FBG, homeostatic model assessment (HOMA‐IR), MDA, hs‐CRP, TC, and LDL. Furthermore, it improved systolic and diastolic blood pressure (Farajbakhsh et al., 2019). It has been shown that in 13 hypertensive patients, administration of capsules with sesamin (60 mg) reduces the level of systolic and diastolic blood pressure (Miyawaki et al., 2009). Among 356 patients with hypertension, it has been shown that the consumption of sesame oil (edible oil, 35 g) for 60 days reduces the blood pressure, TC, LDL, TG, and the amount of TBARS, however, increases the antioxidant activity of SOD, GSH, CAT, GPX, and GSH and also the amount of vitamin C, vitamin E, and β‐carotene (Sankar et al., 2005). In addition, in 30 patients at pre‐hypertension stage, consumption of black sesame meal capsules (2.52 g) for 4 weeks has been reported to reduce the systolic blood pressure, the amount of MDA, and elevates the amount of vitamin E (Wichitsranoi et al., 2011). Similarly, it has been shown that consumption of sesame oil (35 g) could improve flow‐mediated dilatation and reduces the level of ICAM in 30 hypertensive patients (Karatzi et al., 2012, 2013). In addition, the consumption of sesame oil blend (35–40 mL) + nifedipine (20 mg) reduced the systolic and diastolic blood pressure. Also, it lowered the level of TC, TG, and LDL in 300 men with hypertension (Devarajan, Chatterjee, Singh, et al., 2016; Devarajan, Chatterjee, Urata, et al., 2016).

#### 4.7.2 In vivo studies

It has been shown that feeding with sesamin (0.15% w/w) for 4 weeks prevents the cholesterol accumulation in the liver and fought against hypercholesterolemia induced by high‐fat and cholesterol diet in hypercholesterolemic, stroke‐prone/spontaneously hypertensive rats (Ogawa et al., 1995). Similarly, co‐consumption of sesamin (1000 mg) and vitamin E (1000 mg) for 5 weeks reduced the systolic blood pressure, and the level of 8‐hydroxy‐2′‐deoxyguanosine (8‐OHdG) also attenuated thrombotic tendency of cerebral arterioles induced by a helium–neon laser in stroke‐prone spontaneously hypertensive rat (Noguchi et al., 2001). Feeding with sesamin (1% w/w) decreased the systolic blood pressure, the weight of the left ventricle, and vascular hypertrophy in DOCA/salt‐treated two‐kidney, one clip hypertensive rats (Kita et al., 1995; Matsumura et al., 1995, 1998, 2000; Nakano et al., 2004). Moreover, consumption of sesamin (0.1% w/w) for 5 weeks suppressed the increase in the production of vascular superoxide and reduced the systolic blood pressure against hypertension induced by DOCA/salt in rat (Nakano et al., 2002). In addition, oral treatment with sesame oil (0.5 or 1 mL/kg) decreased the systolic and diastolic blood pressure, abnormalities in electrocardiography (ECG), and elevated the level of K^+^ and Mg^2+^. Also, it limited the excretion of K^+^ from urine against hypertension induced by DOCA/salt in rat (Liu et al., 2014). As well, oral treatment with sesamin (>94% purity) elevated the biosynthesis of NO via increasing in the level of phosphorylated eNOS and inhibition of eNOS uncoupling. In addition, it reduced nitrotyrosine, dihydrofolate reductase (DHFR), oxidative inactivation of NO, and the generation of superoxide anion in the aortas of spontaneously hypertensive rats (Kong et al., 2015). Oral administration of sesame protein hydrolysate powder (1 and 10 mg/kg) decreased the level of systolic blood pressure and suppressed the activity of angiotensin I converting enzyme in spontaneously hypertensive rat (Nakano, Kwak, et al., 2006; Nakano, Ogura, et al., 2006). Oral treatment with sesamin (40, 80, and 160 mg/kg) improved the relaxation response of endothelium aorta to acetylcholine, nitroprusside, and increased protein expression of eNOS and the amount of MDA. Also, it reduced protein expression of NADPH oxidase subunits p47 phagocyte oxidase (p47phox) and p22 phagocyte oxidase (p22phox) and fought against the arterial dysfunction in spontaneously hypertensive rats (Zhang et al., 2013). Intragastric injection of sesamin (80 and 160 mg/kg) for 12 weeks reduced the amount of transforming growth factor‐β1 (TGF‐β1), phosphorylated Smad2, protein expression of NADPH oxidase subunits p47phox, and the expression of type I and type III collagen protein. Additionally, it increased the total antioxidant capacity and SOD and fought against myocardial fibrosis in spontaneously hypertension rats (Zhao et al., 2015). As well, oral treatment with sesamin (>94% purity) decreased the blood pressure, the amount of MDA, and the expression of NADPH oxidase subunits p47phox while improving the relaxation response of endothelium aorta to acetylcholine. Also, it increased the biological activity of NO against hypertension and endothelial dysfunction against renovascular hypertension models induced by two‐kidney one clip renal in rats fed with a high‐fat, high‐sucrose diet (2K1C rats on HFS diet) (Kong et al., 2009). Treatment with four demethylated sesamin metabolites (50 μM, orally) elevated the vasodilation response of endothelium in the aorta of rats with hypertension (Nakano, Kwak, et al., 2006; Nakano, Ogura, et al., 2006). It has been shown that oral treatment with sesamin (100 mg/kg) for 3 weeks reduced the hypertrophy of the heart and suppressed fibrosis and inflammation via decreasing the amount of ROS, phosphorylated ERK1/2, and phosphorylated Smad2. The possible mechanism of the protective effect of sesamin to reduce cardiac remodeling induced by transverse aortic constriction in mice appears to be through elevation in phosphorylation of sirtuin 3 (Sirt3) (Fan et al., 2017).

#### 4.7.3 In vitro studies

It has been shown that pretreatment with sesamin (1, 5, and 10 μmoL/L) increased the level of NO, protein, and mRNA expression of eNO and inhibited the level of ET‐1, protein, and mRNA expression of endothelin‐converting enzyme‐1 (ECE‐1). Also, it elevated the biological activity of NOS in HUVECs (Lee et al., 2004).

To conclude, sesame seeds probably show higher protective effects against high blood pressure compared with other oil seeds due to high content of PUFA, such as omega‐3 fatty acids, antioxidant properties, and the lignans, such as sesamin (Vennila, 2017). It increases the biosynthesis of NO, which is one of the factors of vasodilation. Also, it elevates the phosphorylation of Sirt3 that plays a key role in the reduction in cellular ROS levels, and finally inhibition of NF‐ κB, MEK‐ERK1/2, and smad2 signaling pathways. Another possible mechanism is through lowering the protein expression of NADPH oxidase subunits p47phox and p22phox (Figure 1).

### 4.8 Sesame and thrombosis

Various factors, such as free radicals, infection, or trauma inside the blood vessels may harm the walls and initiate the thrombosis cascade (Meade, 1995). The damaged part of the walls of blood vessels acts like a magnet for blood platelets (Kannel, 1997). Raised plasma fibrinogen levels, decreased fibrinolytic activity, and blood clotting time lead to the development of clot formation in atherosclerotic vessels (Smith et al., 1997) (Table 2).

#### 4.8.1 In vivo studies

It has been shown that oral or intra‐arterial treatment with sesamin and sesamolin for 12 weeks has antithrombotic effect against thrombosis induced by He–Ne laser in mice carotid artery (Kinugasa et al., 2011). Also, feeding with sesame or sesame oil (1% w/w) reduced the level of blood clotting fibrinogen and blood clotting factor VII against hypercholesterolemia induced by high‐cholesterol diet in rabbits (Asgary et al., 2013). Due to the high amount of PUFA, sesame oil has the potential to stimulate anticoagulant agents, and reducing blood clots (Jonnalagadda et al., 1996) (Figure 1).

## 5 CONCLUSION

In this review, we collected different in vivo, in vitro, and clinical studies to provide evidences about the role of sesame and bioactive compounds (sesamin and sesamolin) on inflammation and atherosclerosis. Oxidative stress, inflammation, hyperlipidemia, infection, blood pressure, thrombosis, obesity, and diabetes may accelerate atherosclerosis. The possible mechanism of sesame against atherosclerosis appears to be via:

1. Reduction in ROS and oxidative enzymes, such as MDA and TBARS, and increase in the expression of antioxidative enzymes including CAT, SOD, and GPx.
2. Reduction in inflammatory markers IL‐1α, IL‐1β, IL‐6, and TNF‐α, inhibition of the production of COX‐2, arachidonic acid, PGE2, and regulation of the expression of NF‐κB and PPAR‐γ genes.
3. Increase in the expression of ABCG5, ABCG8 genes related to biliary excretion of cholesterol, elevation in lipid oxidation, fat burning, and inhibition in the expression of HMGCR, hepatic FAS, and reduction in the plasma concentration of cholesterol, TG, LDL, and Apo B.
4. Increase in the biosynthesis of NO and phosphorylation of Sirt3 and inhibition of NF‐κB, MEK‐ERK1/2, smad2 signaling pathways, and reduction in protein expression of NADPH oxidase subunits p47phox and p22phox.
5. Reduction in FBS, HbA1c, glucose, glycosylated protein, and increase in insulin and NOS.

Therefore, by suppressing the oxidative stress and inflammation, sesame restricts the VCAM‐1‐mediated adhesion of endothelial cells to activated lymphocytes. Improvement in the metabolism of lipids and glucose, and β‐oxidation of fatty acids is a regulatory mechanism of sesame to fight against the insulin resistance. Sesame is effective in maintaining vascular endothelial function, the balance of adipocytokines, lowering blood pressure, and reducing the platelet aggregation. Of course, all mentioned mechanisms might have synergistic effects in the antiatherosclerotic function of sesame.

## AUTHOR CONTRIBUTIONS

**Elham Hadipour:** Methodology (equal); visualization (equal); writing – original draft (equal); writing – review and editing (equal). **Seyed Ahmad Emami:** Writing – review and editing (equal). **Niloufar Tayarani‐Najaran:** Visualization (equal); writing – review and editing (equal). **Zahra Tayarani‐Najaran:** Funding acquisition (supporting); project administration (equal); visualization (equal); writing – review and editing (equal). Seyed Ahmad Emami was responsible for investigation, methodology, literature searches.

## FUNDING INFORMATION

The study was funded by Research Council of Mashhad University of Medical Sciences, Iran.

## CONFLICT OF INTEREST STATEMENT

The authors report no conflicts of interest in this work. The authors alone are responsible for the content and writing of this article.

## Data Availability

Data wil be available upon request.
